# Spatial Distribution and Temporal Patterns of Cassin’s Auklet Foraging and Their Euphausiid Prey in a Variable Ocean Environment

**DOI:** 10.1371/journal.pone.0144232

**Published:** 2015-12-02

**Authors:** Suzanne Manugian, Meredith L. Elliott, Russ Bradley, Julie Howar, Nina Karnovsky, Benjamin Saenz, Anna Studwell, Pete Warzybok, Nadav Nur, Jaime Jahncke

**Affiliations:** 1 California Current Group, Point Blue Conservation Science, Petaluma, California, United States of America; 2 Department of Biology, Pomona College, Claremont, California, United States of America; 3 Department of Biology, University of South Florida, Tampa, Florida, United States of America; 4 Department of Geography, San Francisco State University, San Francisco, California, United States of America; UC Santa Cruz Department of Ecology and Evolutionary Biology, UNITED STATES

## Abstract

Krill (*Euphausiids)* play a vital ecosystem role in many of the world’s most productive marine regions, providing an important trophic linkage. We introduce a robust modeling approach to link Cassin’s auklet (*Ptychoramphus aleuticus*) abundance and distribution to large-scale and local oceanic and atmospheric conditions and relate these patterns to similarly modeled distributions of an important prey resource, krill. We carried out at-sea strip transect bird surveys and hydroacoustic assessments of euphausiids (2004–2013). Data informed separate, spatially-explicit predictive models of Cassin’s auklet abundance (zero-inflated negative binomial regression) and krill biomass (two-part model) based on these surveys. We established the type of prey responsible for acoustic backscatter by conducting net tows of the upper 50 m during surveys. We determined the types of prey fed to Cassin’s auklet chicks by collecting diet samples from provisioning adults. Using time-depth-recorders, we found Cassin’s auklets utilized consistent areas in the upper water column, less than 30 m, where krill could be found (99.5% of dives were less than 30 m). Birds primarily preyed upon two species of euphausiids, *Euphausia pacifica* and *Thysanoessa spinifera*, which were available in the upper water column. Cassin’s auklet abundance was best predicted by both large scale and localized oceanic processes (upwelling) while krill biomass was best predicted by local factors (temperature, salinity, and fluorescence) and both large scale and localized oceanic processes (upwelling). Models predicted varying krill and bird distribution by month and year. Our work informs the use of Cassin’s auklet as a valuable indicator or krill abundance and distribution and strengthens our understanding of the link between Cassin’s auklet and its primary prey. We expect future increases in frequency and magnitude of anomalous ocean conditions will result in decreased availability of krill leading to declines in the Farallon Islands population of Cassin’s auklets.

## Introduction

In the world’s oceans, climate change has led to widespread shifts in ocean conditions including increases in temperature, decreases in pH, loss of ice and shifts in winds, currents and strength of upwelling events [1]. These changes have led to widespread impacts to the distributions, phenology, and demography of marine species [1]. Under increasingly variable conditions, understanding marine species’ responses is essential to the managers of our ocean resources, allowing them to prioritize areas and species for protection. The objective of this study was to understand how variability in the California Current System (CCS), a highly productive eastern boundary current, impacts the distribution and relationship of an important prey resource, krill (*Euphausiids*), and one of its predators, the Cassin’s auklet (*Ptychoramphus aleuticus*).

In the CCS, recent decades have demonstrated increased variability in ocean conditions along central California (e.g., increased incidence of warm water events) and increased frequency of El Niño-like events [2,3]. Also, the nature of El Niño-like events has changed. Sea surface temperatures have increased since 1995 [4,5,6,7,8] though both warming and cooling are occurring in different locations at different scales. Over the past 30 years, local cooling has been documented along the continental shelf in central California [9,10]. While this upwelling-driven cooling may help maintain healthy food-web dynamics, there has been an increase in the frequency and intensity of extreme events, which has reverberated up to upper trophic predators [2,3].

The Cassin’s auklet is a small, zooplanktivorous, central-place foraging seabird in the Northeast Pacific (Baja California, Mexico to the Alaskan Aleutians) that is capable of diving to depth of up to 40 m [11]. Their reproductive success has been monitored on Southeast Farallon Island (SEFI) since 1972. SEFI is the main breeding colony within the central CCS surrounded by pelagic waters that fall within two National Marine Sanctuaries: Gulf of the Farallones National Marine Sanctuary (GFNMS) and Cordell Bank National Marine Sanctuary (CBNMS). In the past decade extreme variation in breeding occurred: successful double-brooding was recorded in some years while complete colony abandonment was observed in 2005–2006 on SEFI [12,13,14,15,16]. To gain insight into the link between ocean conditions and reproductive success we examined the distribution and abundance of prey in relation to the distribution and abundance of Cassin’s auklets foraging at sea. Establishing the link between foraging seabirds and their environment is critical to understanding how marine food webs are affected by climate change. Seabirds are highly sensitive to localized changes in oceanographic conditions, prey availability, and ecosystem dynamics [17,18,19,20].

The CCS is a highly productive eastern boundary current system dominated by upwelling in the spring and summer months and is home to a large array of marine birds and mammals [14,21,22,23,24]. This upwelling system is replete with mid-trophic level organisms, linking primary producers and upper-trophic level organisms [25]. Euphausiids (family Euphausiidae, known commonly as krill) are marine crustaceans and important mid-trophic level organisms. The euphausiid assemblage in the central CCS is dominated by two species, *Euphausia pacifica* and *Thysanoessa spinifera*, both of which show sensitivity to the strength and duration of upwelling [26]. Cassin’s auklets rely on these mid-trophic level prey and therefore are indirectly influenced by ocean forcing [12,27,28,29,30,31]. Both krill species exhibit diel vertical migration [22,32,33,34], although *T*. *spinifera* has been documented to swarm at the surface during daylight hours within the Sanctuaries [35].

Within the CCS, zooplankton are often aggregated in hotspots, defined as persistent locations where birds and mammals prey on them [36,37,38]. In the central CCS, the greater Gulf of the Farallones region is one of these persistent hotspots [36]. Here, seabirds are associated with specific bathymetric and oceanographic features such as islands and the shelf-break that play a role in aggregating krill [37,38,39, 40,41,42,43]. However, birds’ associations with many of these hotspots vary interannually [38, 43,44] and are often related to the onset, timing, and strength of upwelling.

We hypothesized (1) that the distributions of both krill and Cassin’s auklets are influenced by local bathymetry and local and basin-scale oceanographic conditions, (2) Cassin’s auklet at-sea distribution is correlated with measures of prey biomass in the upper water column, and (3) Cassin’s auklets utilize consistent hotspots where their krill prey are aggregated.

## Methods

To evaluate our hypotheses, we (1) modelled how Cassin’s auklets’ at-sea distributions were related to *in situ* oceanographic, large-scale climate, and bathymetric variables (2004–2013) and (2) examined how their at-sea spatial distributions were related to the distribution of krill detected acoustically (2004–2013). We also (3) examined the zooplankton that Cassin’s auklets collected for their chicks to establish whether what they were taking was the same prey species as what was caught in nets (on SEFI, 1985–2013), (4) used Time-Depth-Recorders (TDRs, 2008–2013) to establish the diving depths of foraging Cassin’s auklets, and (5) collected zooplankton from hoop nets (2008–2011) to determine what was the composition of the acoustically detected prey biomass. Together, these analyses characterize the ecological linkages between Cassin’s auklets and their prey.

### Ethics Statement

All TDR and diet collection work was conducted on the Farallon National Wildlife Refuge with permission and approval of the U.S. Fish and Wildlife Service after thorough review of the project proposal. Auklet diet collection has occurred annually since the 1970s as part of the regular monitoring on the Farallones while TDR deployments have been done annually since 2008. The study was conducted under the terms of Cooperative Agreement #81640-5-J046 (May 2005 –September 2009) and Cooperative Agreement #81640AJ008 (October 2009 –September 2014) and was in agreement with all required state and federal collecting permits. Point Blue Conservation Science’s Federal Banding Permit (#09316) specifically authorizes application of TDRs to Cassin’s auklets and Rhinoceros auklets though field studies only involved Cassin’s auklets which do not have any special protected status. All sampling procedures and manipulations (including deployment/retrieval of TDRs and diet sampling) have been specifically reviewed and approved by U.S. Fish and Wildlife Service and the Farallon National Wildlife Refuge manager. The federal Bird Banding Laboratory (BBL) also reviewed and approved a proposal for the TDR work and subsequently amended Point Blue Conservation Science’s banding permit to specifically include the use of auxiliary markers (including TDR, GPS and GLS tags) on Cassin’s auklets. Personnel concluded that a compatibility determination was not necessary because of the low probability of impact (distress) to the sampled Cassin’s auklets, the small number of birds sampled, the project’s short duration, lack of impact to other island natural resources, and negligible impacts on accomplishing other island duties, personnel resources, and FWS resources.

### Study area and survey design

**Fig 1:**
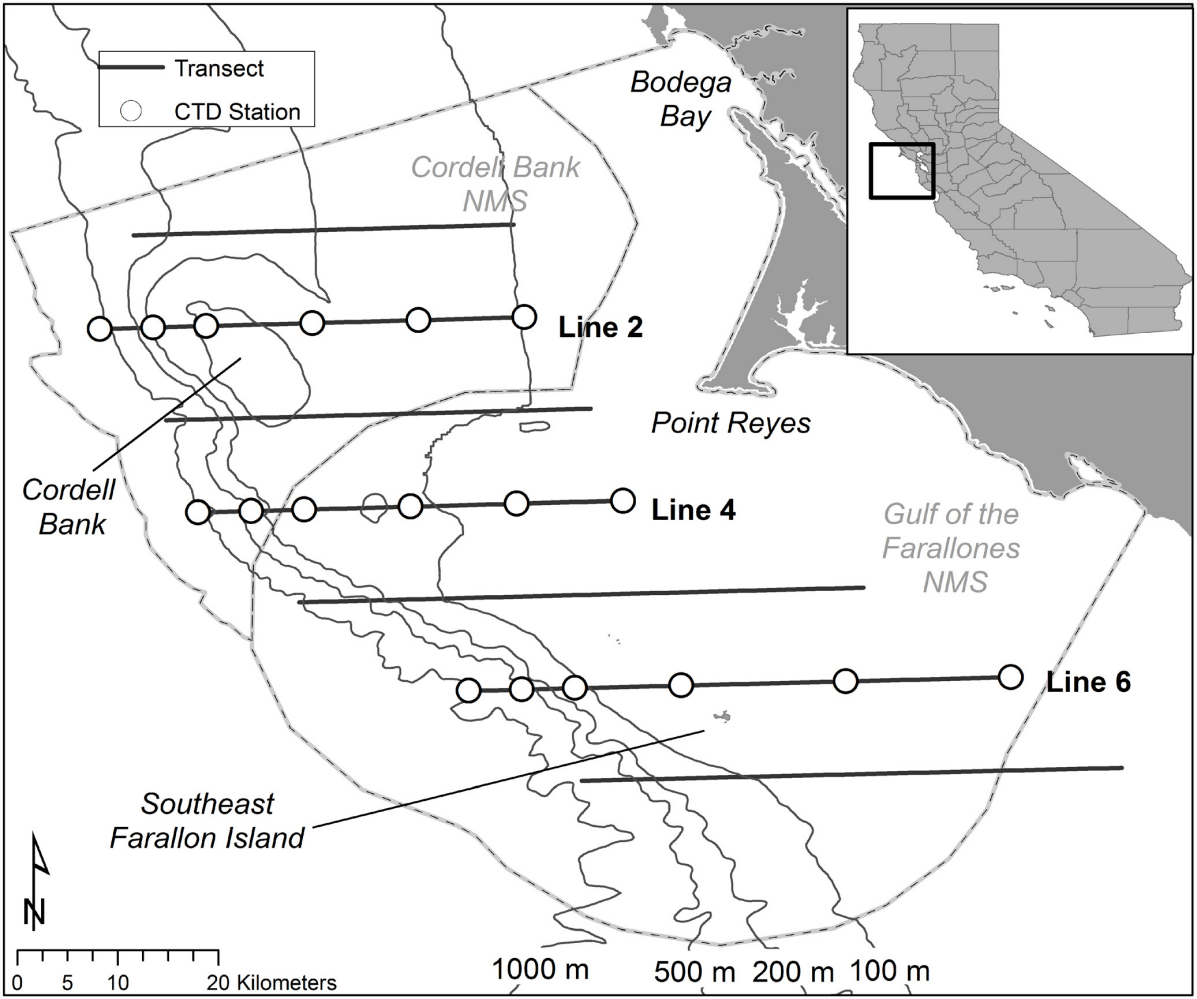
Study area within the Gulf of the Farallones and Cordell Bank National Marine Sanctuaries, California, USA, showing ACCESS program transect lines and oceanographic sampling stations visited for CTD casts and zooplankton tows.

This study used ten years of survey data collected in GFNMS and CBNMS (Fig 1) as part of the Applied California Current Ecosystem Studies (ACCESS) program (www.accessoceans.org), a partnership of GFNMS, CBNMS, and Point Blue Conservation Science. Data were collected between April and October 2004–2013 on three vessels: the *R/V* John H. Martin (Moss Landing Marine Laboratories, Moss Landing, California, USA), the *R/V* McArthur II (National Oceanic and Atmospheric Administration (NOAA), Seattle, Washington, USA), and the *R/V* Fulmar (NOAA, Office of National Marine Sanctuaries, West Coast Region, Monterey, California, USA).

ACCESS conducts multidisciplinary research and monitoring to understand changes in marine wildlife populations and how wildlife responds to changing ocean conditions in the Sanctuaries. ACCESS cruise transect lines primarily ran east-west through the Sanctuaries to sample the area between southern Bodega Bay (38.3°N) and San Pedro Rock (37.6°N); the amount of data collected varied by cruise and year as a function of time constraints and inclement weather. Transect lines 1–7 were used in these analyses and each transect was subdivided into 3 km bins by cruise using methods from prior studies [36,38,42]. Each bin’s centroid was subsequently assigned *in-situ* oceanographic and remotely-collected environmental data, biological data (seabird and krill), and location-related data (e.g., distance to certain features, depth, and contour index). Finally, we discarded end-of-line bins (< 1 km in length) from analyses to avoid introducing errors [38,45,46].

We created a prediction matrix of 1 km^2^ cells based on the Sanctuaries’ boundaries buffered externally by 5 km. We used this matrix to make predictions using selected final models for krill and Cassin’s auklets incorporating environmental information from the 3 km bins. The scale of the matrix allowed for highly detailed visualization of model results.

### Environmental variables: oceanographic variables and oceanographic surfaces

Sea surface temperature (SST), sea surface salinity (SSS), and sea surface fluorescence (SSF) were recorded *in situ* by a thermosalinograph (TSG) located on the hull of each vessel. SSF is a measure of chlorophyll *a*, a proxy for the phytoplankton used as forage by zooplankton species, such as krill. We predicted missing sea surface values, a result of instrument malfunction, using data collected *in situ* at pre-determined, permanent stations using a Conductivity-Temperature-Depth (CTD) profiler (SBE 19plus SeaCAT Profiler CTD with WETStar fluorometer). The closest permanent station to each missing TSG value’s associated 3 km bin was used. Cruises missing all TSG values for a variable were predicted from the CTD station values corresponding to that cruise. To fill in missing TSG values, we used a regression equation incorporating observed TSG values from all ACCESS cruises and the associated CTD values while controlling for month and year. We generated separate models for predicting average fluorescence (R^2^ = 0.63), salinity (R^2^ = 0.88), and temperature (R^2^ = 0.94), all *p* < 0.001, and used those values to complete the dataset.

Using ordinary kriging with optimization, we created interpolated surfaces in ArcGIS (10.2, ESRI Redlands, CA) for the oceanographic variables (SST, SSS, SSF) to predict oceanographic conditions in locations not sampled within and extending 5 km outside the Sanctuaries’ boundaries (*n* = 106). Kriging fits a spatial-dependence model to a set of data and generates a landscape of values of the measured sample points around a predicted location providing a measure of accuracy to the predictive surface [47,48,49]. We employed parameter optimization to standardize the kriging process in order to minimize mean square error for landscape predictions and we applied a default search radius to weight measured locations. Kriging can be sensitive to anisotropy (i.e., can vary with latitude and/or longitude) but there was a spatial trend in some of the oceanographic measurements given the large extent of our study area. To check for raster surface outliers, we compared each variable’s set of surfaces’ average standard errors to ensure all values were within three standard deviations of the mean. We excluded one cruise because of lack of data (*n* = 3 surfaces) and only used values extracted from the study region south of ACCESS transect line 3 to the southern boundary of the region for two others (*n* = 6 surfaces). We then sampled all generated surfaces to extract predicted oceanic variable values for each bin and for each 1 km^2^ prediction cell within the Sanctuaries.

### Environmental variables: climate indices, bathymetric variables, and distance variables

#### Climate indices

In our modeling, we included four variables that fluctuate on multiple timescales and which are known to influence ocean conditions in the CCS: the (1) Pacific Decadal Oscillation (PDO) which is the first dominant mode of variability in sea surface temperature north of 20°N [50]; (2) North Pacific Gyre Oscillation (NPGO) which is the second dominant mode of variability in sea surface height in the northeast Pacific [51]; (3) Southern Oscillation Index (SOI) which is a standardized index based on the observed sea level pressure differences between Tahiti and Darwin, Australia [52]; and (4) Upwelling Index (UI) which is the amount of upwelled water from the base of the Ekman layer per 100 m of coastline (Table 1). We used as a single averaged monthly UI value from two locations along the California coastline (36°N 122°W and 39°N 125°W) to best match the study region. Monthly values of all climate indices were assigned to cruise by month. We included climate lags in monthly increments in modeling to examine the possible influence of ocean conditions up to three months prior to each cruise month.

**Table 1:** Description and ranges of variables used to model krill distribution and Cassin’s auklet abundance using 3 km bins (*n* = 4313).

Variable	Description	Mean ± SD	Min–Max Values
*Oceanographic Variables*			
SST	Average surface temperature (°C)	12.3 ± 1.6	7.9–17.6
SSS	Average surface salinity (psu)	33.4 ± 0.4	29.7–34.3
SSF	Average surface fluorescence (mg / m^3^)	2.3 ± 5.7	- 8.6–28.3
*Bathymetric Variables*			
Distance to land	Distance from bin midpoint to nearest mainland feature (km)	26.6 ± 14.9	0–56.4
Distance to SEFI	Distance from bin midpoint to Southeast Farallon Island (km)	35.3 ± 18.4	0–100.4
Distance to Cordell Bank	Distance from bin midpoint to Cordell Bank (km)	14.5 ± 11.0	0–47.3
Distance to 200 m isobath	Distance from bin midpoint to 200 m isobath (km)	37.8 ± 19.5	0.5–115.5
Average depth	Average depth at bin midpoint (m) (available from California Department of Fish and Wildlife)	- 400 ± 596	- 2719– -1
Contour index	Contour index: (max depth—min depth)/max depth) (unitless) (available from California Department of Fish and Wildlife)	0.12 ± 0.16	0–1
*Climate Indices (present month up to 3-month lag)*			
SOI	Monthly Southern Oscillation Index value(http://www.cgd.ucar.edu/cas/catalog/climind/soi.html)	0.32 ± 1.39	- 2.70–4.30
PDO	Monthly Pacific Decadal Oscillation value(http://jisao.washington.edu/pdo/PDO.latest)	- 0.31 ± 1.03	- 2.21–1.86
NPGO	Monthly North Pacific Gyre Oscillation value(http://eros.eas.gatech.edu/npgo/)	0.57 ± 0.97	- 1.71–2.09
UI value	Averaged Upwelling Index value (36°N 122°W & 39°N 125°W)(http://www.pfeg.noaa.gov/products/PFEL/modeled/indices/upwelling/NA/data_download.html)	174.68 ± 68.32	46.00–339.50
*Effort- or detection bias-related variables*			
Cell Count *	Vertical cells, for which krill were summed (unitless)	30 ± 7	0–95
Sea State **	Observed Beaufort scale conditions +	2.0 ± 0.2	0–6
Swell **	Observed swell height (m) ++	1.0 ± 0.6	0–8
Visibility **	Observer visibility (unitless, dependent on atmospheric conditions e.g. haze, fog, rain)	6.0 ± 0.3	0–9
Time of Day **	Time of completion per bin (HHMM)	1201 ± 429	0608–2005
Cloud Cover **	Observed cloud cover values recorded	7.9 ± 0.7	0–9

All variables were used in both modeling exercises with exception of effort (* used only in krill analysis) or detection (** used only in CAAU analysis) variables.

^+^ 98% of data observed in a 0 to 2 condition;

^++^ 99% of data observed in a 0 to 1 m condition.

#### Bathymetric and distance variables

For each observation point along ACCESS cruise transects and each 1 km^2^ prediction cell midpoint, we calculated distance to important locations and depths. Important locations included nearest mainland feature, the island breeding colony SEFI, Cordell Bank, and the 200 m isobath (or shelf-break). Distances were calculated in ArcGIS from the midpoint of each 3 km bin or 1 km^2^ prediction cell. For each bin and cell, we also calculated average depth using a bathymetric surface (available from California Department of Fish and Wildlife) and generated a contour index value, a metric of ocean floor roughness: (maximum depth—minimum depth) / maximum depth [42].

#### Krill hydroacoustics surveys

Zooplankton was sampled acoustically every 2 s along transects using a Simrad EK60 echosounder. Raw volume backscatter data were post-processed and integrated into 200 m horizontal by 5 m depth bins using Echoview software (Sonardata, Pty. Ltd.). To avoid surface interference, backscatter from 0–5 m were excluded. The level of noise reduction was adjusted by visually comparing echograms until noise at depth appeared equal [53] and time-varied noise was subtracted from each echogram before integration into bins.

Watkins & Brierley [54] found that by selecting for a decibel difference between 120 kHz and 38 kHz of 2–16 dB, portions of the echogram could be classified as Antarctic krill (*Euphausia superba)*. We evaluated the Stochastic Distorted-Wave Bourne Approximation (SDWBA [55,56]) for models of *Euphausia pacifica* generated from images of body shape and published animal densities [57], and found no significant difference between the *E*. *pacifica* and *E*. *superba* models. We therefore used the simplified SDWBA [58] to generate krill acoustic target strengths for 8–30 mm length krill, roughly the size-range of juvenile, immature, and adult stages of the euphausiid species of interest. Based on these target strengths, we classified echogram bins as krill using a decibel difference of 11–19 dB. Echogram bins that fell within the 11–19 dB range were then used as a positive mask over the original 120 kHz echogram data for further processing. This method of classification functions well in single species aggregations; however, in the CCS, there are many more types of zooplankton that may be similar acoustically to krill, adding to classification uncertainty. Furthermore, the sampled area may contain not only other zooplankton but also fish, altering the average target strength sampled by the echosounder. Despite these limitations, we believe the dB difference method of krill classification is more accurate than manually classifying aggregations in the echograms.

The dB differencing operation resulted in the inclusion of some layers we judged not to be krill aggregations, due to either weak scattering (e.g. < -75 dB) or the position and shape of the scattering layer in the water column (e.g. too shallow and/or diffuse). Tucker trawls were deployed at the shelf-break down to 200 m depth on the 3 designated CTD lines to sample the zooplankton and ground-truth the acoustic record. Additionally, we manually included several bins excluded from analysis during the dB difference operation. These bins appeared at the center of dense krill swarms, but were excluded due to a large difference between backscatter at 120 and 38 kHz. We theorize that the most dense krill swarms may not exhibit pure Raleigh scattering at 120 kHz, causing backscatter values that fell outside of the 11–19 dB differencing classification. These manual classification adjustments accounted for less than 0.5% of volumetric acoustic returns ultimately attributed to krill.

Krill biomass (g m^-2^) was calculated by first apportioning the acoustic backscatter to a length-frequency distribution of individual krill determined from trawl surveys concurrent to acoustic sampling. The backscatter was divided by the corresponding individual backscattering cross sections and then multiplied by a length-weight relationship to obtain the mass of krill [53]. The length-weight relationship was derived from sampled individual krill *M*_*L*_ = 2.0 x 10^-4^ *L* ^2^–1.7 x 10^-3^ *L*, where *L* is an individual krill length (mm). Acoustic data were integrated throughout the water column to 30 m depth and binned to 3 km bins to match other datasets (*n* = 3046; maximum krill biomass: 24,628 g m^-2^).

### Cassin’s auklet surveys

Seabird observations were systematically collected during each ACCESS cruise from the vessel flying bridge using standardized strip-survey methods while on effort, defined as transiting a transect line with an observer actively recording seabird observations during daylight hours with vessel speed of ~10 knots [14,59]. Data on factors that affect detection (Table 1) were noted and a single observer recorded encounters of seabirds within a 90° arc from the bow to the vessel’s starboard side and within 50–300 m of the vessel (following methods in Jahncke et al. [14] and McGowan et al. [38]). Seabirds’ behaviors were recorded as flying, foraging, ship-attracted, and sitting. Only actively foraging birds or birds sitting on the water (assuming birds fed or were about to feed in the vicinity of where they were resting) were included in analyses [19]. The total number of Cassin’s auklets counted along each transect line was summed for each 3 km bin and assigned to that bin’s midpoint (*n* = 3879; 3047 zero-count bins, 832 non-zero count bins).

### Modeling: krill and Cassin’s auklets

We used Generalized Linear Modeling (GLM) to examine how krill distribution and abundance were related to Cassin’s auklets at-sea distribution and abundance. We treated krill biomass and seabird counts as separate dependent variables and developed two distinctive predictive models (one for each organism) in the Sanctuaries (STATA version 13.0, StataCorp 2013, Statistical Software, College Station, TX). To predict Cassin’s auklet abundance, we used a zero-inflated negative binomial regression model [60]. This approach is well-suited for count data, and allows for the presence of “false zeroes” [60]. For modeling krill biomass at depths of up to 30m (due to lack of auklet foraging below this depth) we fit a two-part model, using the procedure twopm in STATA 13.0. The first part was a logistic regression model estimated for probability of observing positive value (presence of krill) vs. zero. The second part was a negative binomial model that was conditional on a positive value in the first part of the model. Thus it is a type of hurdle model, allowing one to separately model presence vs. absence from abundance, where present [60]. We used twopm because it allows for a positive outcome that is continuous, since the dependent variable is krill biomass per unit volume, rather than a count.

#### Univariate testing and initial modeling

For krill modeling, we conducted univariate testing of variables of interest (Table 1) for both parts of the two-part model to determine whether relationships were linear or not. We generated a full multivariable model including, for each modeled variable, the most significant functional relationships in each part of the model (determined using Likelihood Ratio Statistics (LRS)). Depending on the variable, we used one of several strategies. Significant quadratic terms (*p* < 0.05) were always included; if the quadratic term was non-significant, the linear form of the variable (provided *p* < 0.20) was included in this initial phase. For distance variables, we chose the most significant of four possible relationships to krill based on *a priori* knowledge: linear, quadratic, logarithmic, or inverse logarithmic. For climate indices, we selected either a no-, 1-, 2-, or 3-month lag index by testing each variable for each of the lag periods and selecting the lag with the greatest significance. For average depth, we chose the most significant of eight possible relationships: linear, quadratic, cubic, logarithmic, quadratic logarithmic, inverse logarithmic, quadratic inverse logarithmic, or square root.

We then included the selected form of all variables in a full multivariate model and used manual backwards stepwise removal until all remaining variables were significant (*p* < 0.05), either in the first or second part of the final two-part model. The exception to this rule was with respect to average depth. For Cassin’s auklets, the cubic transformation was preferred (*p* < 0.0001 for the cubic term). For krill abundance (part 2 of the two-part model), we forced in the cubic term (p > 0.7), to allow for an asymmetric polynomial fit (quadratic transformation alone imposes a symmetric pattern); dropping the cubic term would have yielded an artifactual spatial pattern. Thus, both krill and Cassin’s auklet models allowed for a cubic term for depth.

#### Zero-inflated modeling

For Cassin’s auklet modeling, we first used negative binomial regression to test all variables of interest using the same modeling guidelines outlined above. This regression is recommended for data that have a larger variance than expected from a Poisson-distributed dataset [61,62,63]. However, to account for an excess of zeroes compared with a negative binomial distribution, we used zero-inflated negative binomial regression [60].

Zeroes in count data may reflect observer detection biases and ignoring them can lead to poor modeling of a species’ distribution. Using a model that accounts for both “true zero counts” and “false zero counts” is needed to obtain accurate predictions [61,63]. There are four ways zeroes can arise in ecological data: the species is not present because of an unsuitable habitat; the species is not seen because it does not fill the entire suitable habitat; the species readily occurs in the habitat but is not present in the surveyed location at the time of survey; and the species is present during the survey but individuals are not detected due to observer error [64]. In an example of this last case, a failure to detect an individual might be caused by poor observation conditions that prevent the observer from seeing the bird even though it was present.

For both the krill and Cassin’s auklet datasets, variables were included to standardize sampling efforts. As a measure of effort in krill modeling exercises, we used the total number of acoustically-sampled vertical cells within each bin. An offset of the logarithm of the area surveyed within each bin was used for the Cassin’s auklet dataset [38,45]. We tested variables affecting detection probability (i.e., variables used to account for excess of recorded zeroes) in the Cassin’s auklet modeling (Table 1). Bias variables of statistical significance (*p* < 0.05) were left in the model using previously mentioned manual backwards step-wise removal. Finally, we used the Vuong test statistic (STATA version 13.0, [65]) to test if using the zero-inflated negative binomial regression was superior to the standard negative binomial regression [60].

Once we generated final models, we examined potential interactions between year and specific variables based on *a priori* knowledge. We tested for interactions of year with UI, salinity, and temperature for krill and with UI and salinity for Cassin’s auklets and built models incorporating significant interactions, if any, as determined by the likelihood ratio test. We also considered allowing for multiple interactions in a final model and report the significance of those interactions with respect to the likelihood ratio statistic. Variance inflation factor (VIF) testing for multicollinearity [63] was conducted among variables in the final models.

#### Model validation

We validated model fit using k-fold cross validation (*k* = 10, with 20 replicate runs) based on the predictions to the 3 km bins. One at a time, each one of the 10 subsets was used as a “test” dataset and the other nine were combined and used as a “training” dataset. For each run, we calculated a median result; we repeated the procedure 20 times and calculated a median of the 20 medians. We compared the final model pseudo R^2^ value with the median pseudo R^2^ value and reported the comparison. A model validation pseudo R^2^ value within 40% of the final model pseudo R^2^ value was considered a minimally adequate model fit [36].

### Mapping predators, prey, and their overlap

**Fig 2:**
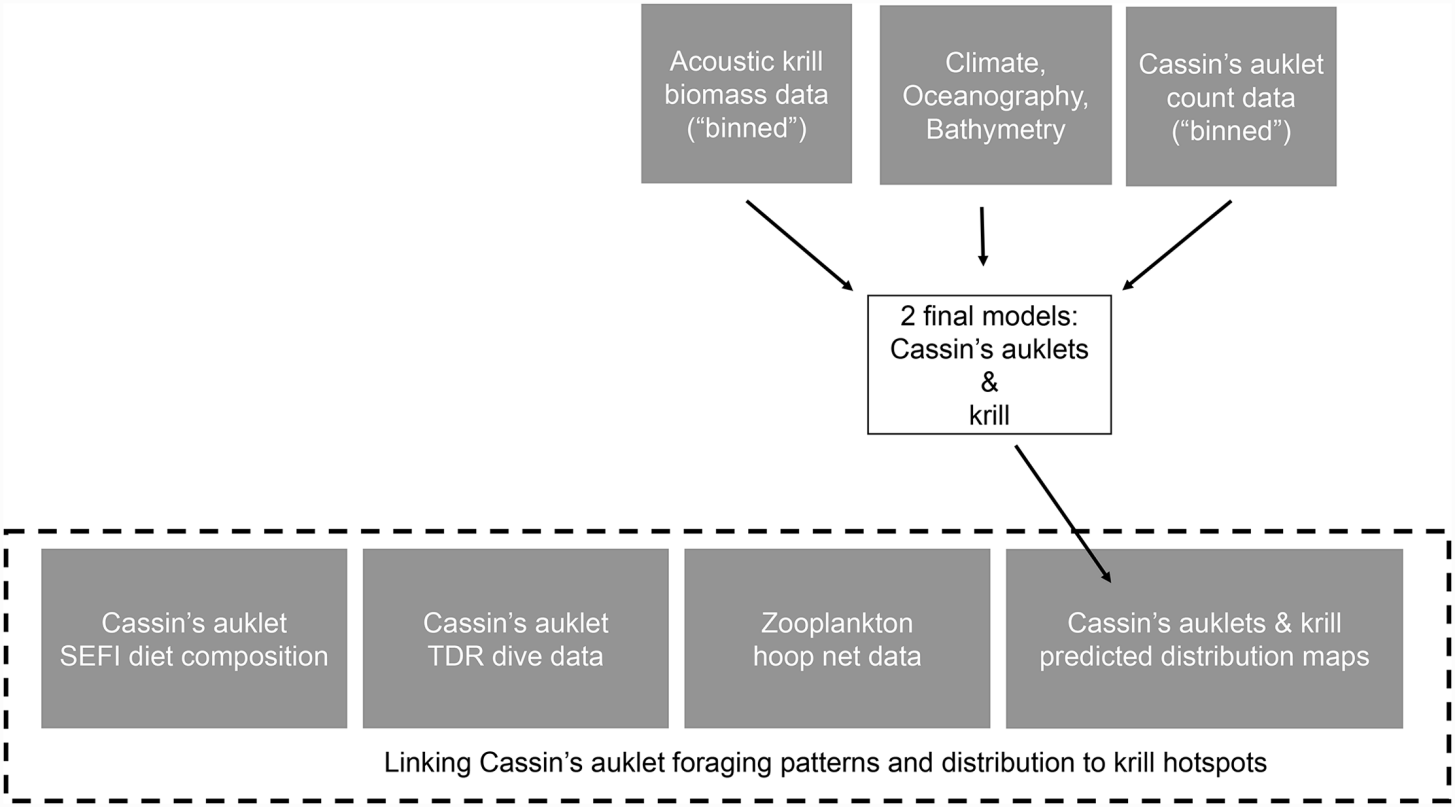
Schematic study modeling methodology showing binned and environmental data, predicted to 1 km^2^ prediction cells, linked with Cassin’s auklet diet data, Cassin’s auklet TDR dive data, and zooplankton tow samples from the upper water column.

We predicted predator (Cassin’s auklet) and prey (krill spp.) distributions in each 1 km^2^ cell using model results and inputs that included observed oceanographic variables (collected during surveys), climate indices (collected remotely), and bathymetric- and distance-related data from 3 km bins (Fig 2). We averaged spatial occurrence predictions separately by month (across all years 2004–2013) and by year (May through September) for each cell of the prediction matrix. We excluded April because it was not sampled as frequently as other months during the study period. We mapped each month or year independently and scales were chosen using percentiles of predicted values from the full predicted monthly or annual dataset to allow for same-scale comparison for each species’ maps. We identified areas of high use habitat for both individual models and combined species in all-inclusive hotspot maps. We mapped areas of co-occurrence, examining the locations of top 5-, 10-, 20-, and 30-percentile habitat use by both krill and Cassin’s auklets.

### Cassin’s auklet diet data

We determined the diet of Cassin’s auklets feeding chicks by capturing adults returning to SEFI at night and collecting regurgitations intended to be fed to their young. Cassin’s auklets carry partially digested prey to their chicks in a sublingual pouch [66]. Adult Cassin’s auklets were captured by hand as they returned to the colony at night. 10 random diet samples were collected each week throughout the chick-rearing period (May to July / August) for 1985 to 2013, excluding 1992. Immediately upon capture, the Cassin’s auklet was inverted with its head over a sterile Whirl-Pak specimen bag and collected samples were frozen until the diet composition analysis could be performed. In most cases, diet samples were obtained when the birds spontaneously regurgitated the contents of their sublingual pouch. If spontaneous regurgitation did not occur, we attempted to induce regurgitation by gently massaging the sublingual pouch for a few seconds. Birds were subsequently released on the ground where they were initially captured immediately after a sample was obtained. See Sydeman et al. [12,18] and Abraham and Sydeman [31,67] for details on diet collections and analysis. For this paper, we show prey composition as the annual proportion of the taxa in the diet, as determined by number. While proportion by number inflates the importance of smaller prey items, we used this measure as we had counts of prey items for all years, while we only had wet weights beginning in 1994 (Point Blue, unpublished data). Results using wet weights were similar but are not reported as they require assumptions about prey masses. We used one-way ANOVA to test for differences in proportion of euphausiids vs. all other taxa (arc-sin transformed) found in the diet in each sample amongst years.

### Cassin’s auklet dive records

We used TDR records to provide justification for use of an acoustic record in the upper 30 m of the water column. Cassin’s auklets at SEFI in the Farallon National Wildlife Refuge were outfitted with TDRs between 2008 and 2013; procedures followed those of Karnovsky et al. [68]. TDR deployment was conducted concurrently with ACCESS cruises to fully explore the link between birds, their prey, and ocean conditions. The TDRs (CEFAS Technology Limited, United Kingdom) were approximately 8 mm diameter x 31 mm length in size, weighed 2.7 g in the air (or 1.5% of the average initial mass of instrumented birds), and had a cross sectional area of 0.50 cm^2^ (or between 1 and 2% of instrumented birds’ cross sectional area). They were programmed to record temperature and pressure every 5 s while the bird was on the surface or at the colony and every 0.5 s whenever the bird dove below 1.5 m. Instruments were cylindrical in shape and had a rounded tip to minimize water resistance [69]. The instrument was attached to the ventral side of the bird by gluing it to the feathers of the belly using a few drops of Loctite adhesive. We deployed TDRs (*n* = 73) on adult Cassin’s auklets which were brooding young chicks in artificial nest boxes. We attempted to balance the sex ratio to account for any potential biases. We used one-way ANOVA to test for differences in dive depths amongst years.

To evaluate potential negative impacts on Cassin’s auklet breeding biology of TDRs, we compared reproductive success (RS; chicks fledged) of long-term study birds (non-instrumented; Point Blue, unpublished) with RS of pairs where one adult was fitted with a TDR on SEFI between 2008 and 2013 using logistic regression controlling for year as a categorical variable. Additionally, we compared the growth rates of chicks of long-term study birds and pairs where one adult was fitted with a TDR on SEFI for 2008 using a 2-tailed t-test.

### Zooplankton tow data

In order to evaluate the potential available prey for Cassin’s auklets, we examined zooplankton data from daytime surface tows. We sampled zooplankton during ACCESS cruises July 2004 through September 2011 in daytime hours (0600–1800 hours) using a 1 m diameter hoop net fitted with 333 μm mesh. Oblique tows (50 m to the surface) were performed at each CTD station, towing the net at 1–2 knots for about 10 min. Volume filtered was estimated from a mechanical flowmeter (General Oceanics model 2030RC) attached to the opening of the hoop net frame. The contents of each tow were preserved in 10% formalin solution and stored in 1 L plastic jars.

Preserved samples were identified and enumerated at the Institute of Ocean Sciences, British Columbia, Canada. Zooplankton identification protocols were consistent with previous zooplankton analyses [70]. In brief, entire samples were scanned for abundance of large and/or rare taxa. Using a Folsom splitter, the sample was quantitatively sub-sampled for counts of small abundant zooplankton taxa. Total counting effort was ≥ 400–500 individuals per sample, sufficient to give an expected subsampling error of ≤ 20% for the dominant species [70,71]. We used a chi-square test (χ^2^, Pearson’s) to determine differences of adult and juvenile krill counts in hoop net samples amongst years.

## Results

### Acoustically-sampled krill: habitat association and predicted distribution

We found that all tested variables were significant predictors for the amount of krill in one or both parts of the two-part model. SST, SSS, and SSF all significantly predicted presence for krill. All climate indices (SOI, PDO, NPGO, and UI) were significant, with varying month lags. Bathymetric and all distance variables were also significant predictors of the amount of krill observed (Table 2, S1 Table). No significant multicollinearity was found between variables in the final model (VIF < 10). Cross validation (*k* = 10) pseudo R^2^ was within 40% of krill final model pseudo R^2^ (Table 2).

**Table 2:** Krill and Cassin’s auklet model results with transformation (L = linear, Q = quadratic, C = cubic) and coefficient sign for significant variables. Predictive models used a two-part model combining logistic and negative binomial regressions for krill biomass (g m^-2^; *n* = 3046) and a zero-inflated negative binomial regression for Cassin’s auklet counts (birds km^-2^; *n* = 3879). The bold variables represent significant (p < 0.01, Likelihood Ratio Test) interactions of that variable with year and coefficient sign refers to the main effect without any interactions.

Variable	Krill biomass (g m^-2^)–part 1 (logit)	Krill biomass (g m^-2^)–part 2 (glm)	Cassin’s auklet counts (birds km^-2^)
*Model of Best Fit*			
Temperature	**L (+)** *	**Q (-)** ***	
Salinity	L (+) ***	Q (-) *	**Q (-)** *
Fluorescence	Q (-) ***	L (+) ***	
Distance to land	L (+) **	L (+) *	
Distance to SEFI		L (-) *	L (+) ***
Distance to 200 m isobath		Q (-) ***	Q (-) ***
Distance to Cordell Bank	L (+) ***	Q (+) ***	
Average depth		C (-)^‡^	C (+) ***
Contour index		Q (-) *	Q (-) **
SOI	L (-) ***, 0 mo. lag		Q (+) ***, 1 mo. lag
PDO	L (-) ***, 1 mo. lag		Q (-) ***, 3 mo. lag
NPGO		Q (+) ***, 3 mo. lag	
UI value	**Q (+)** *** **, 3 mo. lag**	**L (+)** *** **, 3 mo. lag**	L (-), 2 mo. lag *
Cell count (effort)	L (+) note not sig.	L (+) ***	N/A
Sea state	N/A	N/A	L (+) **
Visibility	N/A	N/A	L (+) **
Model χ2 (*df*)	1191.09 (48)		818.39 (35)
Model *p*	< 0.0001		< 0.0001
Vuong test for zero inflation	N/A		0.0043
*Crossfold validation (k = 10)*, *20 simulations*			
Final Model R^2^	0.2823		0.0898
Validation R^2^	0.1256		0.0401
Ratio of Validation R^2^: Final Model R^2^	44.50%		44.65%

*p*-values:

‡ ≥ 0.05;

* < 0.05;

** < 0.01;

*** < 0.0001.

**Fig 3:**
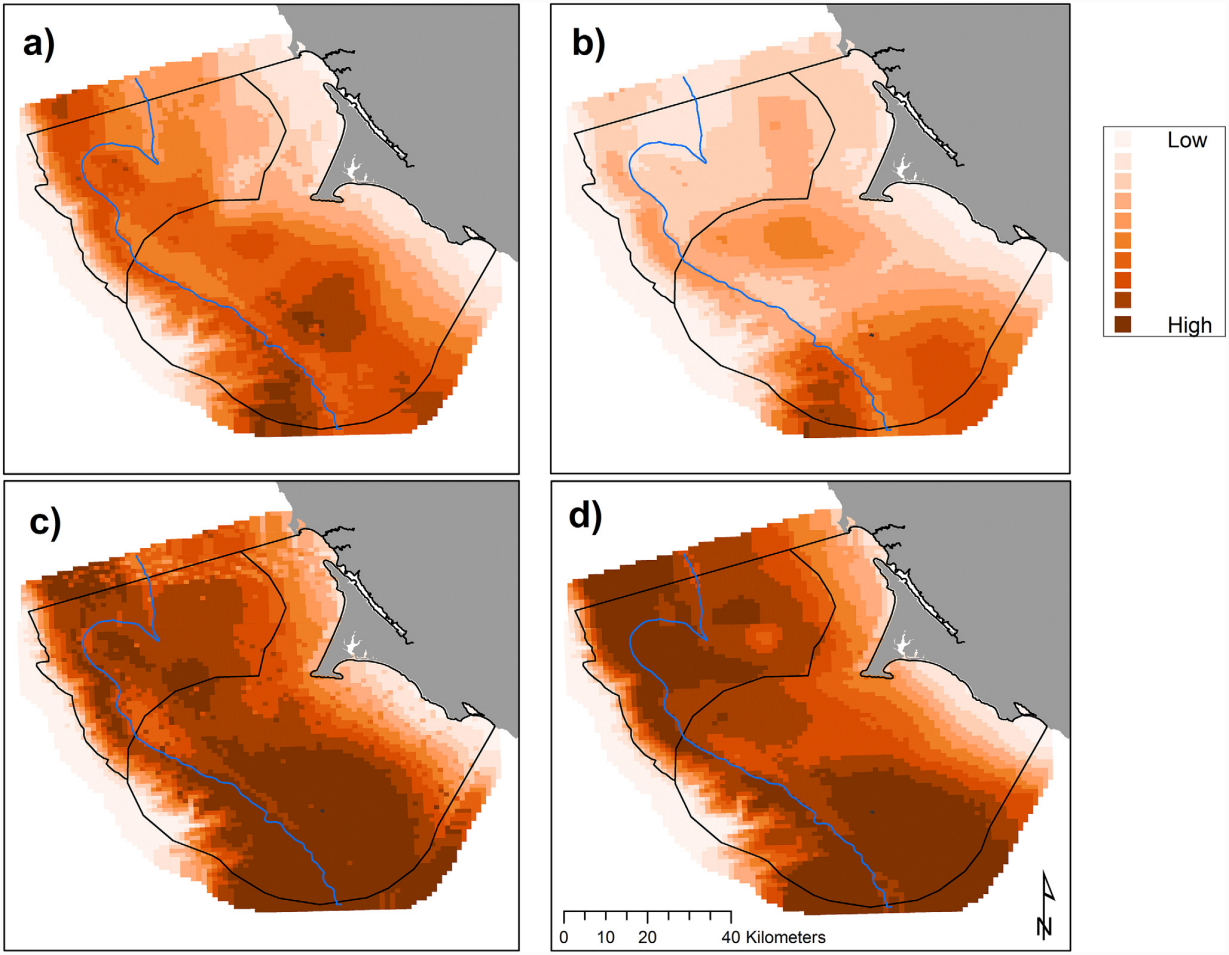
Predicted krill biomass (g m^-2^), 2004–2013: variations in modeled habitat use for May (a, predictions averaged over *n* = 8 years), June (b, *n* = 7), July (c, *n* = 7), and September (d, *n* = 8); each gradation represents a decile, monthly mean values with all years pooled. The 200 m isobath and boundaries of National Marine Sanctuaries included in blue and black, respectively.

**Fig 4:**
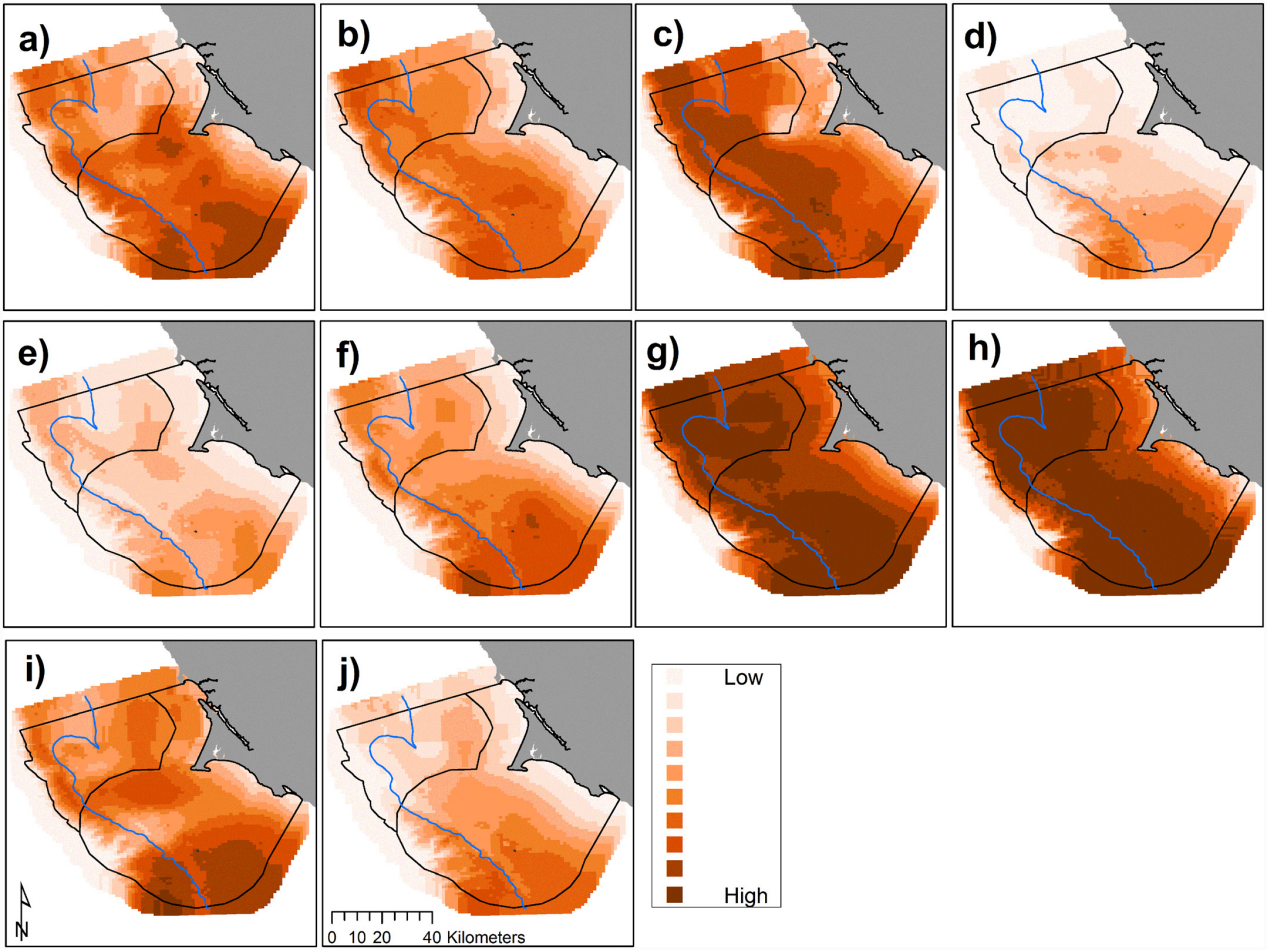
Predicted krill biomass (g m^-2^), 2004–2013 (a-j, sequentially): variations in annual modeled habitat use (predictions averaged over *n* = 3 months except 2010, *n* = 4, and 2013, *n* = 2); each gradation represents a decile, annual mean values with all months pooled per year. The 200 m isobath and boundaries of National Marine Sanctuaries included in blue and black, respectively.

**Fig 5:**
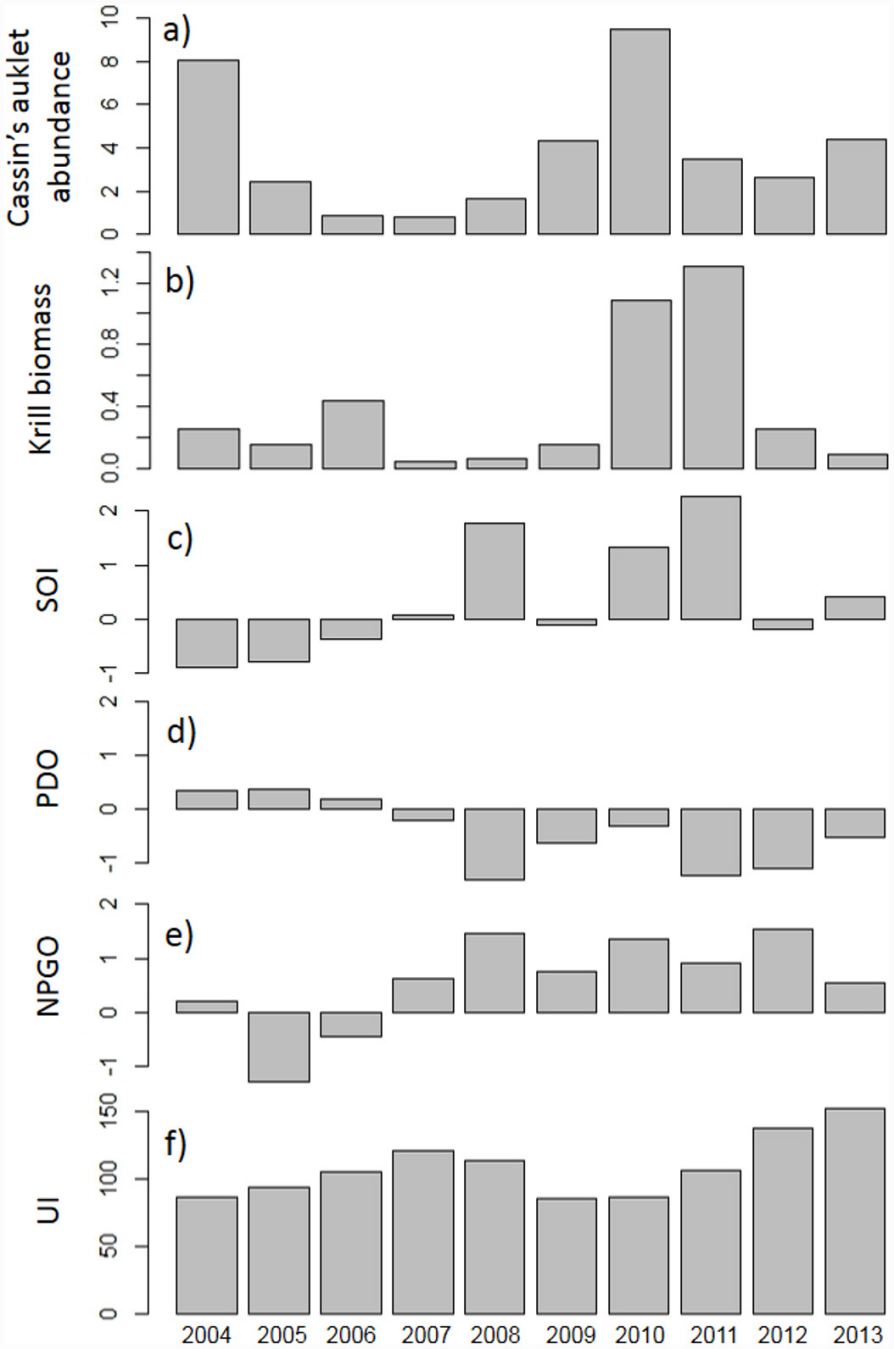
Annual variation in predicted (a) Cassin’s auklet abundance (birds km^-2^) and (b) krill biomass (g m^-2^), yearly averaged values for (c) the Southern Oscillation Index (SOI), (d) the Pacific Decadal Oscillation (PDO), (e) the North Pacific Gyre Oscillation, and (f) Upwelling Index within the Gulf of the Farallones and Cordell Bank National Marine Sanctuaries by year (2004–2013) with all months pooled.

**Fig 6:**
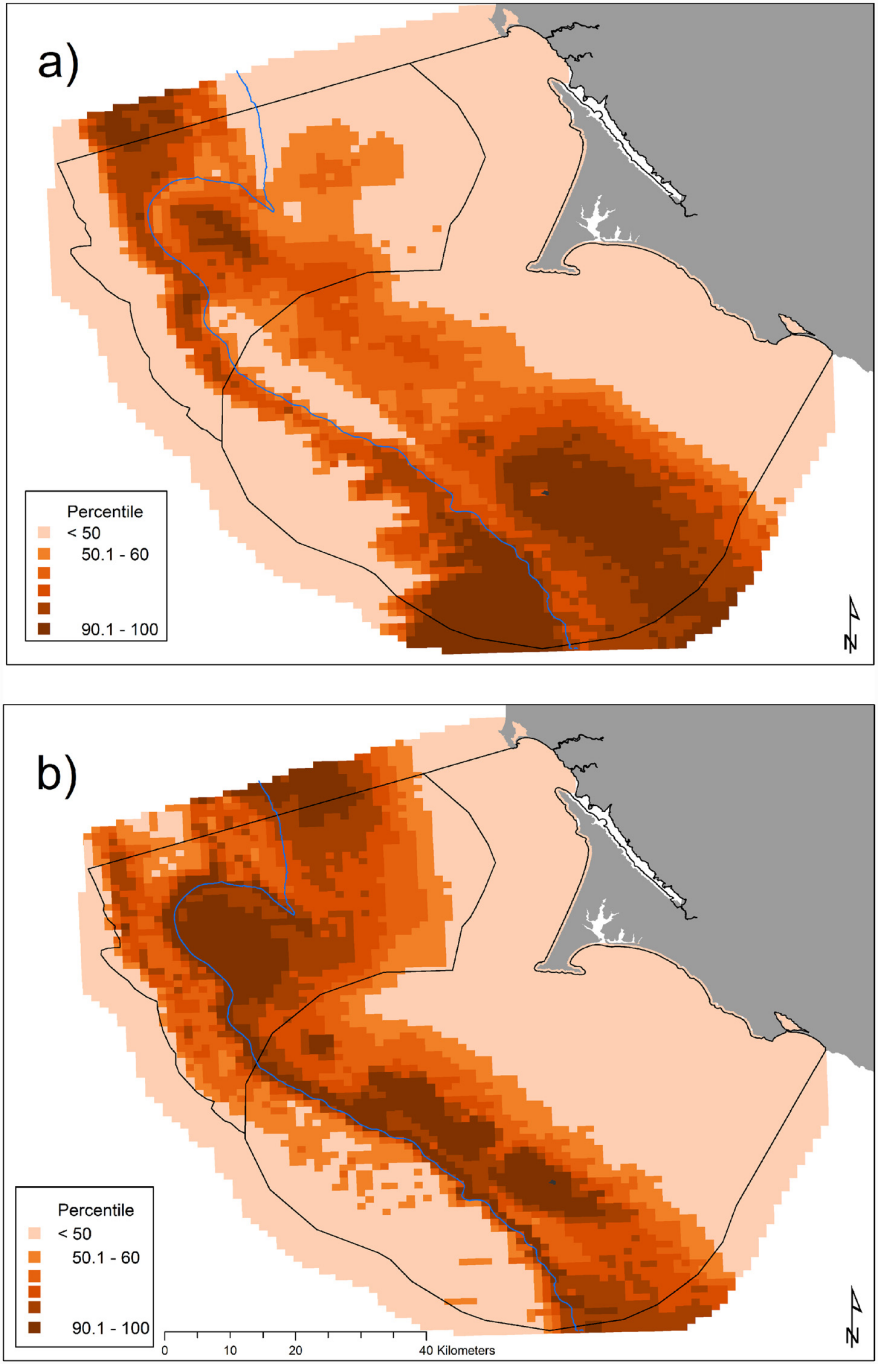
Predicted (a) krill biomass (g m^-2^) and (b) Cassin’s auklet abundance (birds km^-2^), 2004–2013: top habitat use model applied across all months and years; each gradation represents a decile. The 200 m isobath and boundaries of National Marine Sanctuaries included in blue and black, respectively.

The influence of oceanic processes varied among years with respect to the amount of krill present in the upper 30 m as shown by the inclusion of two significant year-interaction terms for both parts of the two-part species model: 3-month lag UI (LRS = 437.17, *df* = 26) and SST (LRS = 414.63, *df* = 35). The influence of processes driven by changes in climate on predicted krill biomass varied among indices: the localized influence of upwelling (UI (LRS = 547.19, *df* = 29)) was an order of magnitude greater than climate variables that operate on a basin-wide scale [PDO (LRS = 26.0, *df* = 1), SOI (LRS = 50.12, *df* = 1), and NPGO (LRS = 74.38, *df* = 2)]. Krill habitat use varied across months (Fig 3) and across years (Figs 4 and 5) with June and 2007–2008 exhibiting the least predicted krill biomass within the Sanctuaries. Krill biomass varied with climate processes (Fig 5). Krill hotspots were consistently predicted across all months and years in the areas surrounding SEFI, southeast of SEFI, and Cordell Bank (Fig 6).

### Cassin’s auklets: habitat association and predicted distribution

Sea surface salinity (SSS) was significantly associated with Cassin’s auklet distribution along with three climate indices with varying time lags: SOI, PDO, and UI. The influence of SSS on individuals also varied annually; we included the interaction of that oceanographic variable with year (LRS = 102.43, *df* = 9). Overall, Cassin’s auklets were found in regions of higher salinity. Additional significant predictive variables were distances to SEFI and the 200 m isobath, contour index, and average depth (Table 2, S2 Table). Birds were found closer to the shelf break in areas of higher bathymetric relief and in association with shallower depths near SEFI. From all tested detection variables, only sea state and visibility significantly contributed to the Cassin’s auklet zero-inflation model (*p* < 0.01). The influence of climate on predicted Cassin’s auklet counts varied among indices: those that operate on a basin-wide scale [PDO (LRS = 89.94, *df* = 2) and SOI (LRS = 74.50, *df* = 2)] were an order of magnitude greater than that of a localized process [UI (LRS = 5.69, *df* = 1)]. No significant multicollinearity was found between variables in the final model (VIF < 10). Cross validation (*k* = 10) pseudo R^2^ was within 40% of Cassin’s auklet final model pseudo R^2^ (Table 2).

**Fig 7:**
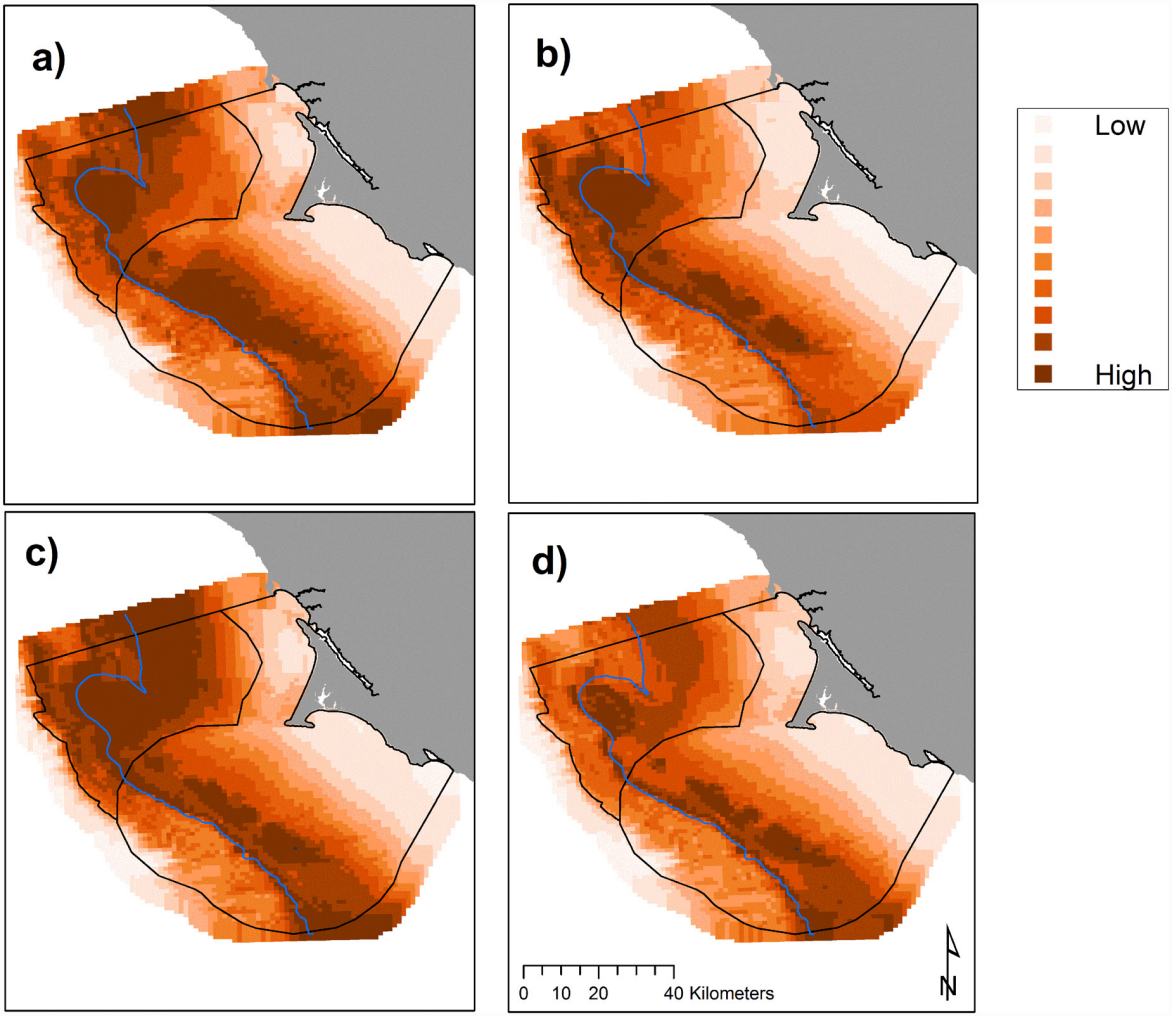
Predicted Cassin’s auklet abundance (birds km^-2^), 2004–2013: variations in modeled habitat use for May (a), June (b), July (c), and September (d); each gradation represents a decile, monthly mean values with all years pooled. The 200 m isobath and boundaries of National Marine Sanctuaries included in blue and black, respectively.

**Fig 8:**
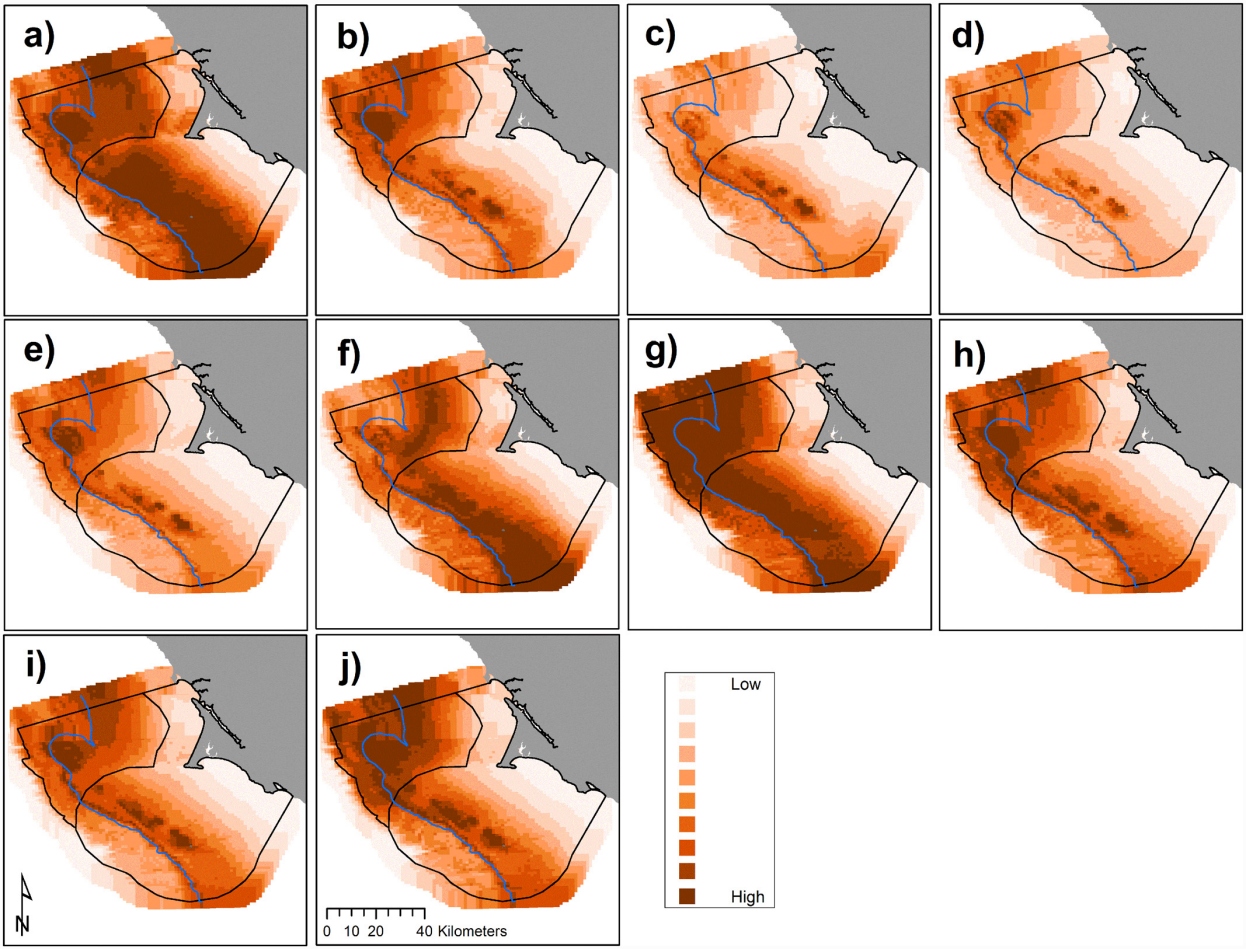
Predicted Cassin’s auklet abundance (birds km^-2^) by year, 2004–2013 (a-j, sequentially): variations in annual modeled habitat use; each gradation represents a decile, annual mean values with all months pooled per year. The 200 m isobath and boundaries of National Marine Sanctuaries included in blue and black, respectively.

We found that hotspot locations of Cassin’s auklets varied across months (Fig 7) and across years (Figs 5 and 8). Like krill, predicted values of Cassin’s auklet counts varied with climate processes (Fig 5). Auklets were present in the study area during all surveys reflecting use of the Sanctuaries across all surveyed months. Auklet occurrence was predicted to be high in 2004 and 2010, but low in 2007–2008. Cordell Bank and the area to the east of the 200 m isobath along the Farallones Escarpment were highlighted as areas where birds consistently foraged (Fig 6).

### Predator and prey co-occurrence

**Fig 9:**
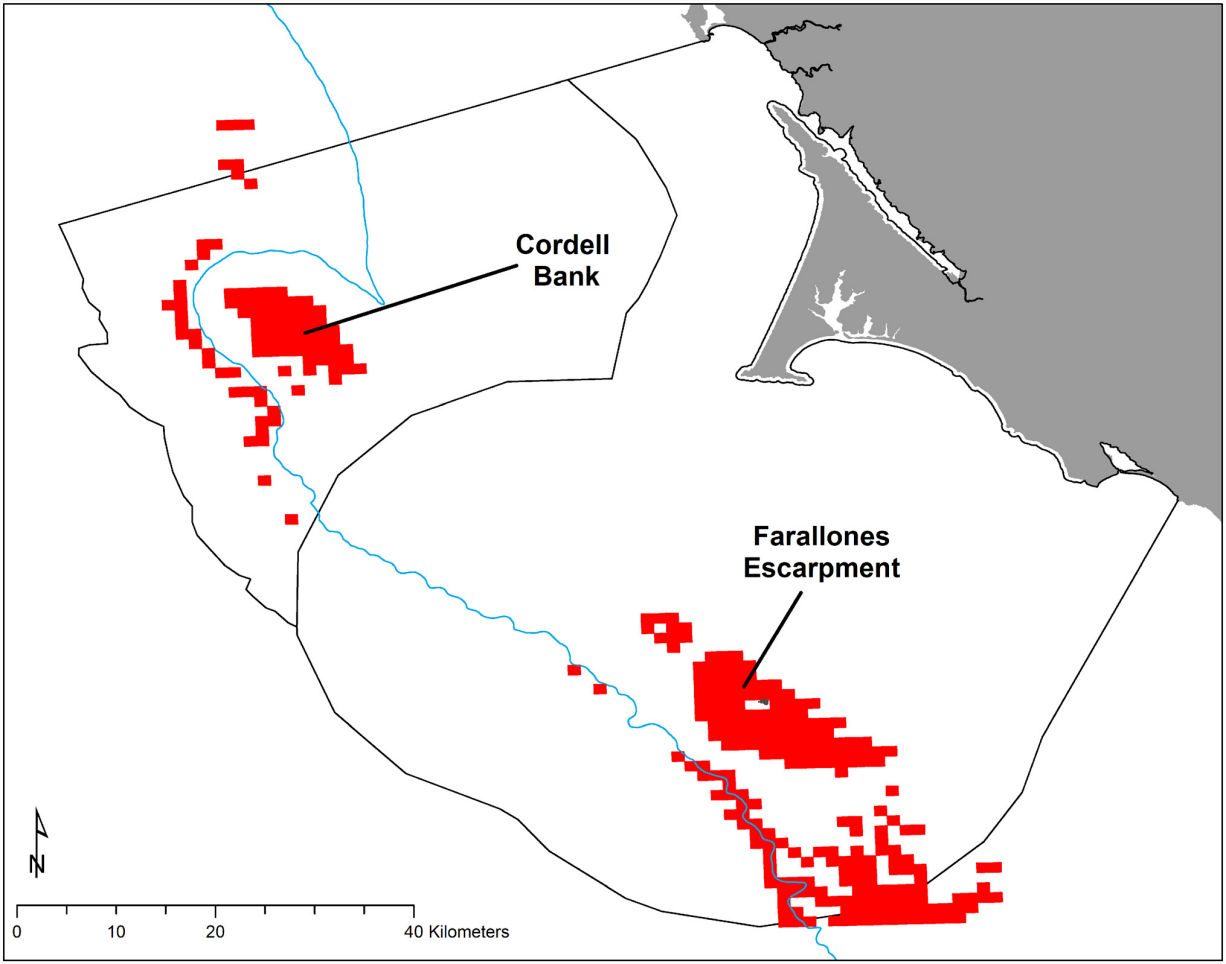
Intersection of high predicted spatial co-occurrence in red for krill and Cassin’s auklets combined over May-July and September 2004–2013 at top 20% habitat use for each predictive model. Consistent high use areas (Cordell Bank and the Farallones Escarpment) are noted. 200 m isobath and boundaries of National Marine Sanctuaries included in blue and black, respectively.

**Fig 10:**
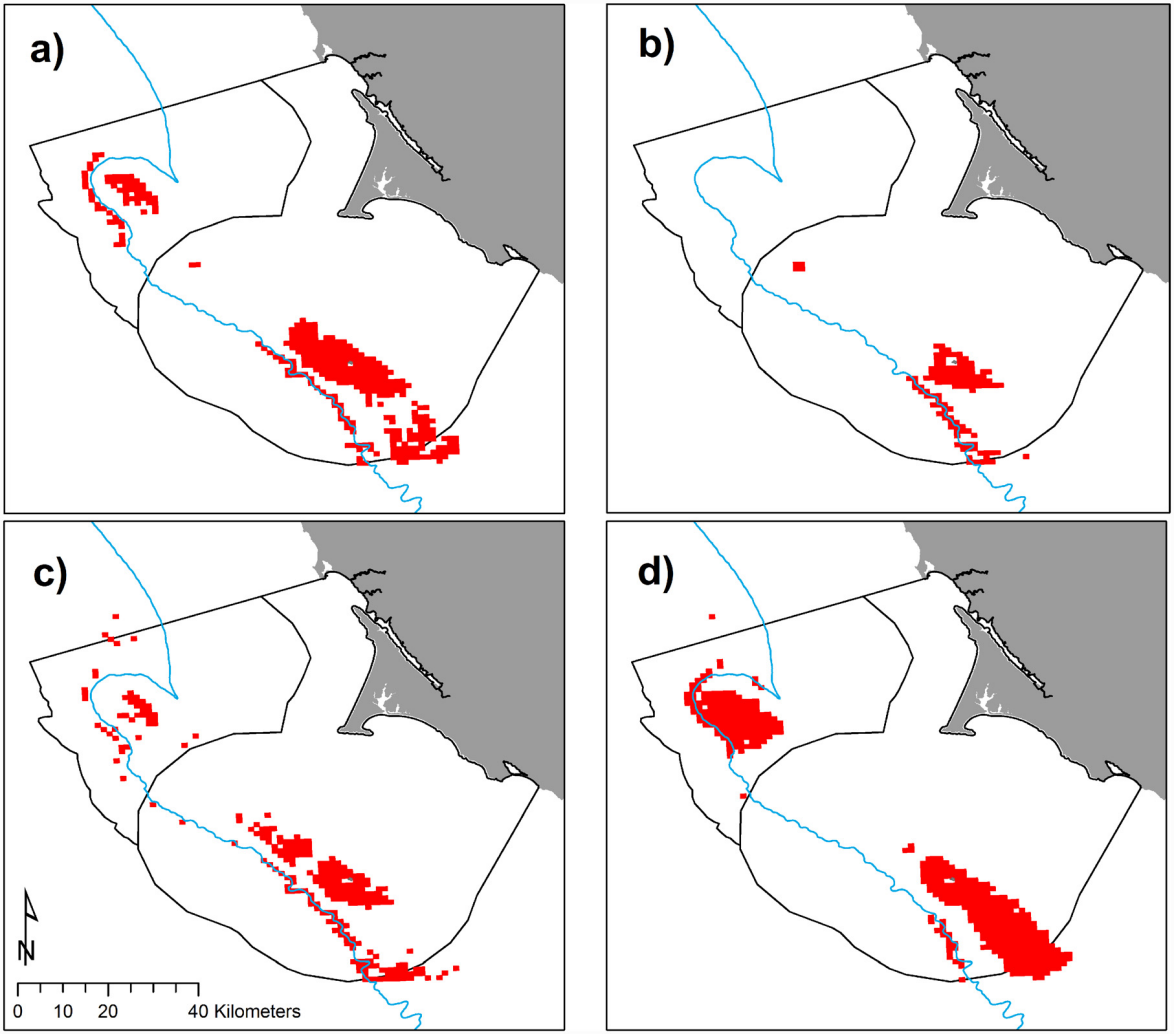
Intersection of high predicted spatial co-occurrence in red for krill and Cassin’s auklets for May (a), June (b), July (c), and September (d) at top 20% habitat use for each predictive model. 200 m isobath and boundaries of National Marine Sanctuaries included in blue and black, respectively.

**Fig 11:**
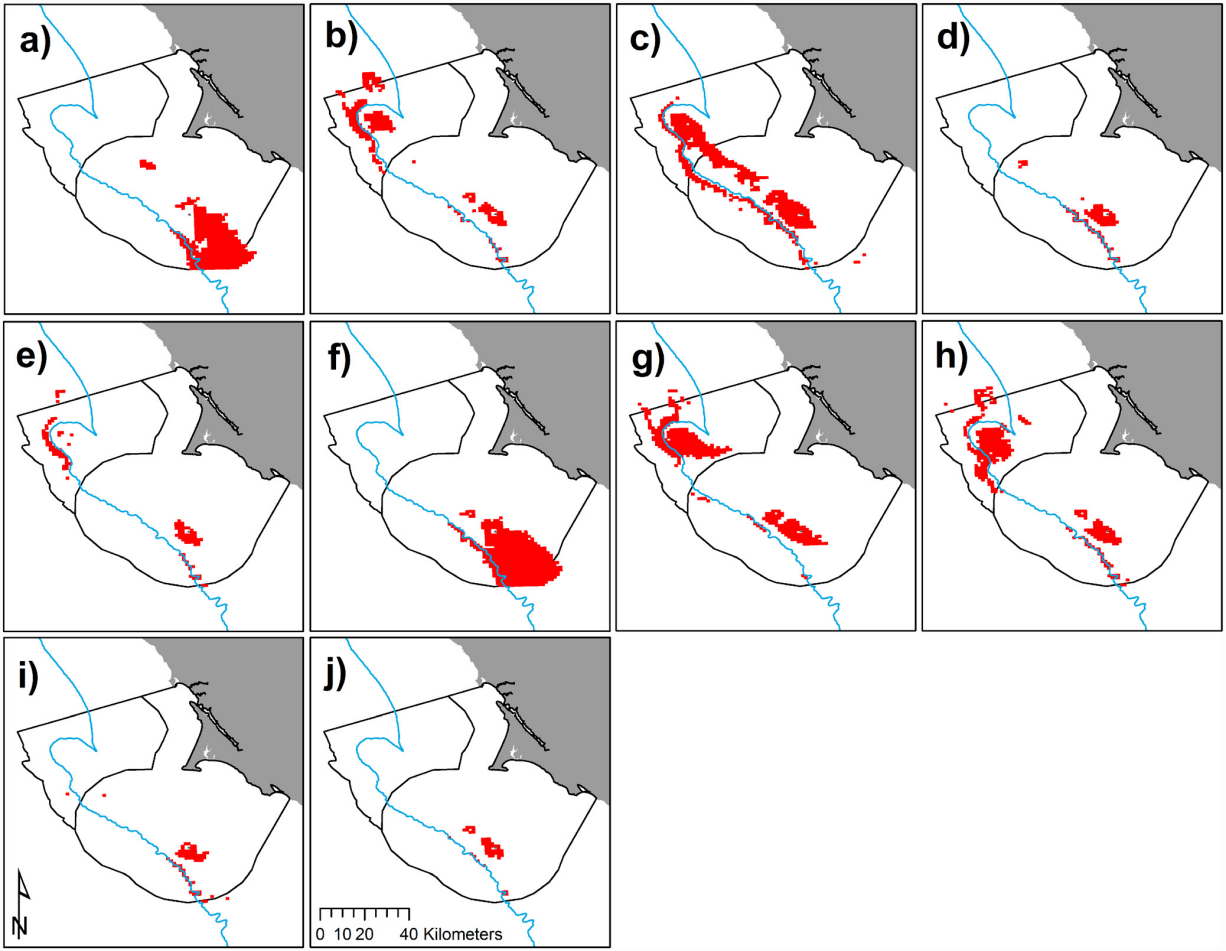
Intersection of high predicted spatial co-occurrence in red for krill and Cassin’s auklets 2004–2013 (a-j, sequentially) at top 20% habitat use for each predictive model. 200 m isobath and boundaries of National Marine Sanctuaries included in blue and black, respectively.

We examined areas of high predicted abundance for both krill and Cassin’s auklets across all months and years, combined. Areas of most likely spatial co-occurrence (as indicated by top 20% use by both species) were the Farallones Escarpment surrounding SEFI and over Cordell Bank (Fig 9); however, these areas varied both annually and by month (Figs 10 and 11).

### Cassin’s auklet diet

**Fig 12:**
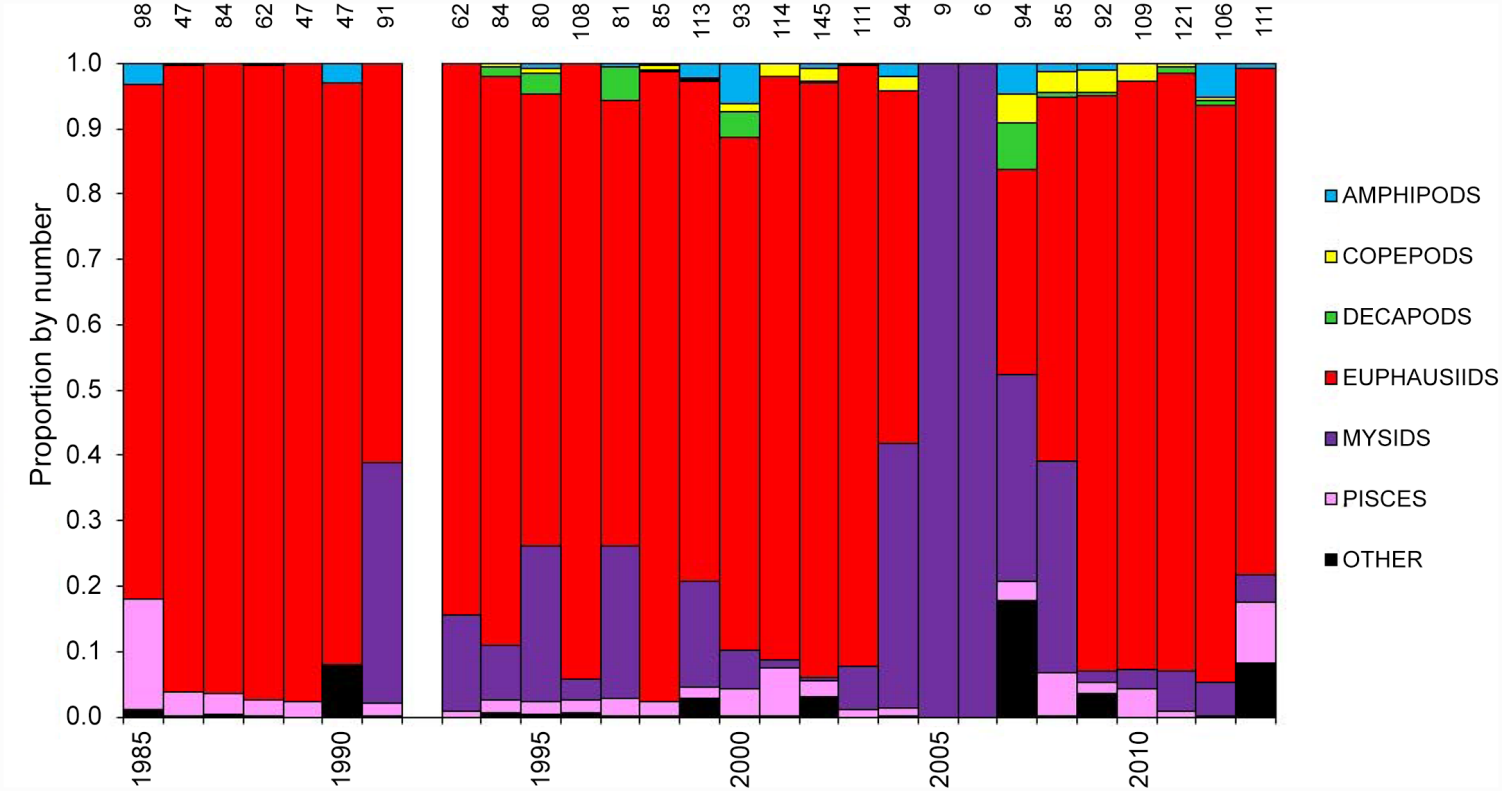
Annual proportion of major zooplankton taxa, by number, in the chick provisioning diet of Cassin’s auklets on Southeast Farallon Island, 1985–2013. Numbers above the bars indicate sample sizes.

Euphausiids dominated the diet of the Cassin’s auklet in 22 of the 27 years of prey composition data from SEFI (Fig 12). We found significant differences in euphausiid consumption among all years analyzed (ANOVA F_(27,2351)_ = 20.40, p = 0.000). While sample size varied among years (Fig 12), the few samples collected in 2005–2006 (*n* = 9 and *n* = 6, respectively) contained almost entirely mysids.

### Cassin’s auklet diving behavior

The maximum dive depth recorded by a Cassin’s auklet was 39.5 m, although most dives were shallower with an overall median dive depth of 7.9 m (Table 3). We found significant differences in dive depths among the 6 years of data collection (ANOVA F_(5,57510)_ = 1371.64, p = 0.000). While 55% of tagged birds’ records (*n* = 40) had at least one dive greater than 30 m, 99.5% of all dives across years (*n* = 57,516) were shallower than 30 m (*n* = 57,216).

**Table 3:** Summary of TDR records (*n* = 73) overall and by year (2008–2013).

	Overall	2008	2009	2010	2011	2012	2013
Mean Dive Depth (m)	9.5	7.3	8.7	11.4	16.4	13.6	8.8
Standard Error	0.03	0.07	0.04	0.09	0.15	0.09	0.05
Maximum Dive Depth (m)	39.5	39.5	38.5	36.2	38.5	29.9	38.1
Median Dive Depth (m)	7.9	4.4	7.1	10.3	16.7	13.8	7.6
Standard Deviation	6.53	6.23	5.98	7.05	8.14	5.08	5.66
Total Dives (*n*)	57,516	8,988	20,119	6,570	2,854	3,279	15,706
Dives < 30.01 m (*n*)	57,216	8,968	20,086	6,501	2,716	3,279	15,666
Dives < 30.01 m (%)	99.5	99.8	99.8	99	95.2	100	99.8
Birds with Maximum Dive Depth < 30m (*n*)	33	10	11	3	0	5	4
Birds with Maximum Dive Depth > 30m (*n*)	40	5	12	7	5	0	11

Attaching TDRs to Cassin’s auklets had no significant influence on breeding success (chicks fledged per breeding pair) or growth rates of chicks where one adult was fitted with a TDR (chick weight change g day^-1^). Between 2008 and 2013, breeding success of TDR-outfitted birds compared to long term study birds was higher for 5 of the 6 years (Point Blue, unpublished data; TDR birds: success = 0.887, SD = 0.318, n = 106; Non TDR birds: success = 0.780, SD = 0.318, n = 255). In 2008, there was no statistical difference between chick growth rates for the two groups (p = 0.86); chicks from nest sites with non-instrumented parents grew at an average of 5.25 g day^-1^ (SE = 0.10; n = 40) while chicks from nest sites with one adult fitted with a TDR grew at an average of 5.37 g day^-1^ (SE = 0.074; n = 10).

### Zooplankton tows

**Fig 13:**
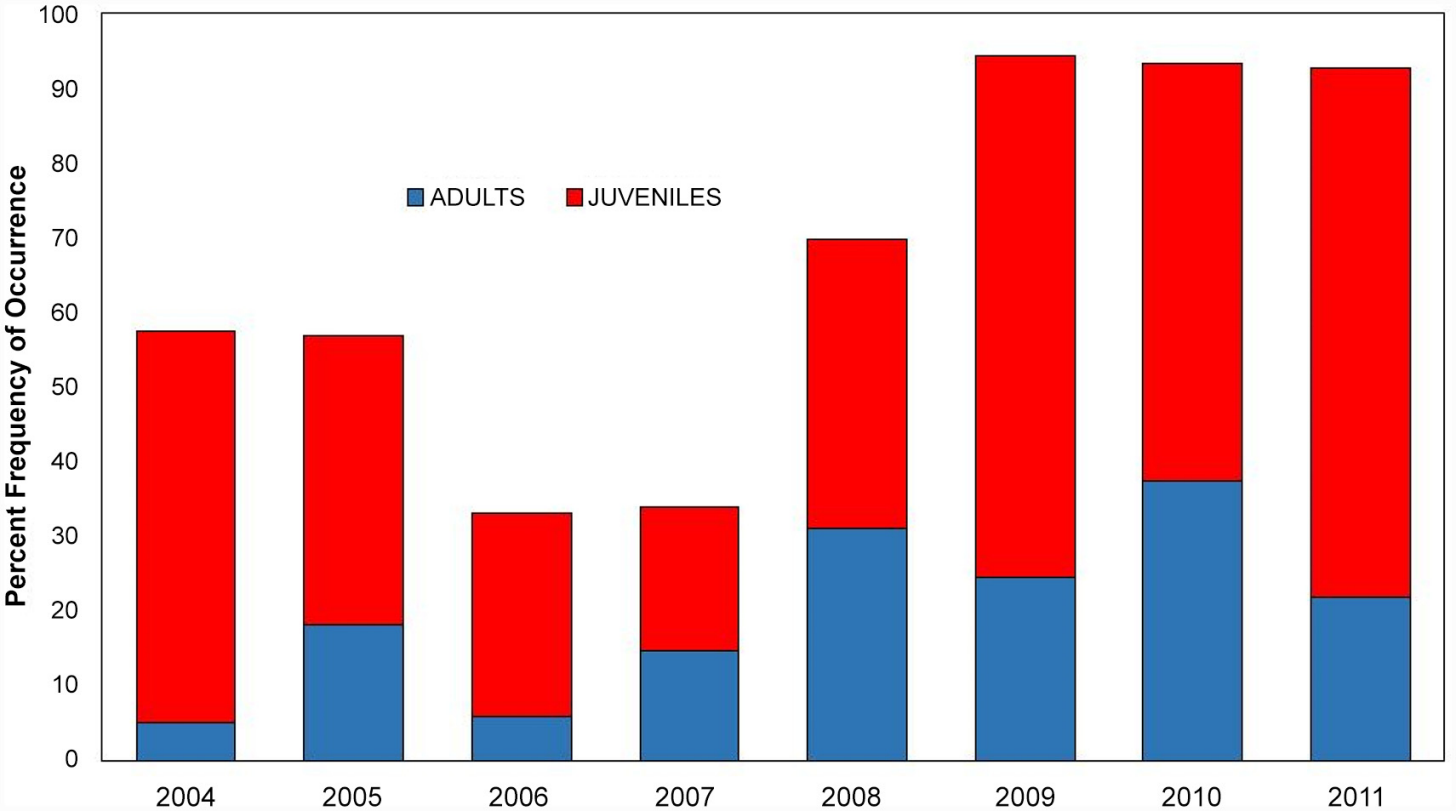
Percent frequency of occurrence of E. pacifica and *T*. *spinifera* adults and juveniles in surface hoop net samples (2004–2011).

We found that adult krill (combined *E*. *pacifica* and *T*. *spinifera*) were present in 20.38% of the surface hoop net samples, and juveniles were present in 42.68% of samples in the eight year time series (Fig 13). We found a significant difference in krill occurrence in hoop net samples for both adults and juveniles among the eight years of data (adults: χ^2^ = 42.3451, *df* = 7; juveniles: χ^2^ = 62.9727, *df* = 7). Abundance (shown through percent frequency of occurrence) varied among years, especially for juveniles; adults ranged from 5.00% (in 2004) to 37.33% (in 2010), and juveniles ranged from 19.12% (in 2004) to 70.73% (in 2011). These tows suggest krill were frequently available in the upper 50 m of the water column.

## Discussion

Within the years of this study we found variability in oceanic conditions, krill distribution and abundance, and responses of the Cassin’s auklet. Both krill and Cassin’s auklets aggregated along the shelf break in areas of productive upwelled waters; their distribution was influenced by local ocean processes and basin-wide climate conditions. Though hotspots varied temporally, we generally found persistent predicted krill habitat in the Cordell Bank region and around SEFI over the study period (Figs 3, 4 and 6). Krill were concentrated away from the mainland, close to the Farallones Escarpment and Cordell Bank in areas of higher bathymetric complexity. Our results corroborate previous studies that found a large center of abundance of *E*. *pacifica* off central California [72], often in the form of long narrow bands along the outer shelf [43]. Additionally, other research found large densities of auklets within 25 km of SEFI and along the shelf break [43]. These areas have been proposed by other studies to be krill hotspots important to seabirds [38,43,73] and new studies (including the present one) have corroborated the high availability of krill in these areas [37].

Foraging Cassin’s auklets, like krill, were associated with Cordell Bank, the shelf break, and the Farallones Escarpment, and we found seasonal and interannual variability associated with these features (Figs 6, 7 and 8). Seasonal variation showed that the shelf break west of SEFI was consistently important during all surveyed months, while Cordell Bank was most important during the post-breeding season. Auklets’ annual use of the shelf-break region west of SEFI and the Farallones Escarpment also varied. Cordell Bank was important to auklets in half of the surveyed years, notably 2005–2006 and 2010–2011 (2005 displayed particularly low krill density while 2010 and 2011 displayed high density).

Localized and basin-scale processes driven by changes in climate affected the abundance of Cassin’s auklets and krill biomass within the Sanctuaries. Our models indicated that predicted krill abundance was associated with waters upwelled 3 months prior to the prediction month. Previous studies on euphausiids within the CCS have found significant associations between krill and UI at different lags (30-, 60-, or 365-day), revealing the dependence of adult krill survival and development upon both short- and long-term ocean climate conditions [31,67]. Additionally, Cassin’s auklet abundance was predicted to be higher with greater sea surface salinity, signifying the importance of upwelled waters.

Cassin’s auklets are sensitive to changes in prey availability which is directly driven by changes in climate [74]. Previous modeling efforts [38] suggested Cassin’s auklets are more strongly influenced by larger-scale ocean forcing compared with local conditions. Conversely, our models show krill biomass to be more strongly influenced by local conditions than basin-scale climate. This finding suggests that predators and prey respond to ocean conditions at differing spatial scales likely associated with disparate individual ranges. Warm El Niño years have been intensifying [4] and a major reduction in zooplankton biomass has been recorded [75]. Recent research has shown how the Cassin’s auklet’s response to different climate variables has changed over time [14,15,67,76]; this may be due, in part, to the increase in frequency of a different type of Pacific warming event (termed El Niño modoki), which is caused by a disruption in the normal atmospheric circulation [77]. An increase in frequency and magnitude of these unproductive conditions will negatively impact marine species on multiple trophic levels [2,3].

While Cassin’s auklet diets from 1985 through 2013 were generally comprised of krill, non-euphausiid food items were present during anomalous years. For example, when combining diet results from 2005–2008, mysids made up >50% of chick diet composition, yet in all other years mysids comprised less than 50% of the chick diet. While mysids and decapods are the dominant shrimp-like crustaceans at depth (>500 m below the surface), krill are one of the most abundant crustaceans in the pelagic environment [78]. Additionally, although few diet samples were collected in 2005 and 2006, composition was almost entirely mysids. The lack of euphausiids in the diet during the unprecedented breeding failures of 2005 and 2006 points to the importance of krill to this species’ breeding success. From 2007 through 2013, the proportion of euphausiids in the auklet diet has increased and breeding success has been high.

Past studies on the Farallon Islands identified *Euphausia pacifica* and *Thysanoessa spinifera* as the main prey consumed by Cassin’s auklets, making up ~80% of the prey items identified [12,18,29,67,79]. When considering the krill portion of the auklet diet, these two species comprise ~96% of the krill observed in the diet in all years (Point Blue, unpublished data). Between 1985 and 2002, the cumulative adult stages of both euphausiid species comprised 82% of euphausiids eaten by Cassin’s auklets. Only in two years (1995 and 2014) did younger age classes (juvenile and immature stages) outnumber adults in the euphausiid portion of the diet, and in only two other years (2000 and 2009) did adult stages make up less than 70% of the euphausiid portion of Cassin’s auklet diet. In the remaining 23 years, adult euphausiids made up between 72% and 100% of euphausiids consumed by Cassin’s auklets. The larger mature stages of these species are the dominant age class consumed (adult lengths: 11.0–25.0 mm for *E*. *pacifica*, 16.0–25.0 mm for *T*. *spinifera*), while smaller juveniles and immature stages of both species are eaten to a lesser degree (juvenile/immature lengths: 5.0–11.0 mm for *E*. *pacifica*, 6.0–16.0 mm for *T*. *spinifera*; [78]).

A study on the Farallones revealed that Cassin’s auklets generally feed on less energy-rich *E*. *pacifica* during pre-breeding and the early part of the breeding season after which there may be a switch to more energy-rich *T*. *spinifera* [31,80]. Stomach samples from Cassin’s auklets on SEFI revealed different krill species were consumed during different upwelling scenarios; *E*. *pacifica* was consumed during times of upwelling relaxation (e.g., warmer surface water), while *T*. *spinifera* was the main prey during times of increased upwelling (e.g., cooler surface water [30]). Abraham and Sydeman [67] documented that Cassin’s auklet productivity correlated with chick growth rate, and growth rate was correlated with proportion of *T*. *spinifera* in the Cassin’s auklet diet between 1996–2001, suggesting the importance of this krill species to auklet reproductive success and chick development. A warming period in the CCS from the 1970s to the late 1990s, marked by more intense warm-water events, was associated with a decreased overall zooplankton biomass and a decline in many seabird populations [4,75,81].

Dive records from TDR-instrumented Cassin’s auklets revealed significant use of the upper water column. Burger and Powell [11] showed similar results, with auklets expending most of their foraging efforts in shallower portions of the upper water column (surface to 30 m) targeting euphausiid prey. Dives of different depths are a signature of alcid foraging behaviors associated with searching for prey [82,83,84]. We found adult krill at or near the surface in daytime towed hoop net samples across sampling years and Cassin’s auklet dive depths showing use of the upper water column by foraging, both interesting findings given later life stages of krill are known to exhibit diel vertical migration as a predator-avoidance mechanism [85].

Cassin’s auklets foraging for krill in the upper water column varied on a year-by-year basis. From 2010–2011, auklets were capturing abundant krill within the Sanctuaries as indicated by hoop net sampling; predicted krill density was also high. Conversely, in 2008, krill was less available for auklets as indicated by predicted densities and hoop net sampling. The story is less clear in 2009, when krill availability in hoop net samples and diet samples was high (just as in 2010–2011), but predicted krill density showed average levels.

Two years of particular interest were 2005 and 2010: 2005, when the first complete abandonment by Cassin’s auklets of the SEFI breeding colony was observed, and 2010, the year of the highest recorded breeding success to date. Low breeding success has been documented in strong El Niño years (e.g., 1983, 1992, and 1997), but in such years complete abandonment was not observed, as observed in 2005 and 2006 [31,67]. In 2005 and 2006, chick diet was highly anomalous: these are the only years exhibiting an absence of euphausiids in the chick diet; in the previous 19 years, euphausiids comprised at least 50% of items fed to the chick. In 2005, modeled krill abundance was low (Fig 5), though not extremely so, suggesting that the nature of the timing or spatial location of krill may have limited the ability of Cassin’s auklets to provide krill to their chick. Low krill abundance in turn was associated with strongly negative NPGO in that year. Other studies in the CCS have demonstrated the effect of a chronological mismatch between auklets’ breeding demands and plankton productivity [86,87].

In spring 2010, central CCS waters demonstrated a quick recovery to normal upwelling conditions following a mild El Niño event [16]; predicted Cassin’s auklet and krill biomass data reflected productive conditions. Krill biomass was higher still in 2011 than in 2010, yet reproductive success was lower in 2011 than in 2010. Euphausiid abundance in the southern CCS, off Baja California, though not directly spatially comparable, was relatively high towards summer and fall 2010, possibly a consequence of a productive cool-water La Niña event [16]. Again, 2011 was a stronger La Niña year than 2010, yet, as noted, reproductive success, while good that year, was not as high as 2010. The reproductive strategy of Cassin’s auklets, and attendant success, reflects the complexities of timing and location of prey availability as well as the condition of parents during the course of the breeding season [88].

The broader implications of this study are to highlight the value of hotspots along shelf breaks for krill predators in all upwelling systems, and that daytime feeding on diel migrating krill may still play a major role in the diet of these predators. In addition, although persistent krill habitat was identified, characteristics of predator prey relationships will likely vary with local and wide scale oceanographic conditions. For example, a shift was documented in trophic level patterns when seabird density in the southern California CCS changed, corresponding to the variability in krill and forage fish abundance [89]. Elements like the optimal foraging theory and predator response to prey shift and availability should be considered when managers are preparing for the impacts of climate changes across varying geographic scales.

Our study highlights persistent areas of high use by foraging Cassin’s auklets around Cordell Bank and the Farallones Escarpment, areas in which krill consistently occur. Changes in the local oceanography and basin-scale climate can lead to weakened or delayed upwelling and warmer ocean temperatures, thus decreasing prey availability. Diet samples confirmed a largely euphausiid-dominated diet among Cassin’s auklets that foraged in the upper water column. Despite diurnal migrations, some adult and late juvenile krill remain in the upper water column during daylight hours, when and where they are preyed upon by Cassin’s auklets. A projected increase in frequency and magnitude of anomalous ocean conditions may affect the long-term stability of the SEFI Cassin’s auklet population, as krill becomes less available, causing reduced breeding success and adult survival [13]. More broadly, krill predators in other systems may face similar impacts with oceanographic changes.

## Supporting Information

S1 Table Coefficients, standard errors, z values, p values, and associated 95% confidence intervals for all quantitative variables (including year and interactions with year) for the two-part model combining logistic regression and negative binomial regression for krill. Model output assumes base year is 2004.
(DOCX)

S2 Table Coefficients, standard errors, z values, p values, and associated 95% confidence intervals for all quantitative variables (including years and interactions with years) for the zero-inflated negative binomial regression model for Cassin’s auklets. Model output assumes base year is 2004.
(DOCX)
