# Supplementary material for: Spatial Distribution and Temporal Patterns of Cassin’s Auklet Foraging and Their Euphausiid Prey in a Variable Ocean Environment
Source: PLoS One. 2015 Dec 2;10(12):e0144232. doi: 10.1371/journal.pone.0144232 (PMC4668006; doi:10.1371/journal.pone.0144232)
Supplement: S1 Table — Model output assumes base year is 2004. (DOCX) [file pone.0144232.s001.docx]

S1 Table. Coefficients, standard errors, z values, p values, and associated 95% confidence intervals for all quantitative variables (including year and interactions with year) for the two-part model combining logistic regression and negative binomial regression for krill. Model output assumes base year is 2004.

| Two-part model |  |  |  |  |  |  |
| --- | --- | --- | --- | --- | --- | --- |
| Log pseudolikelihood | = -9847.109 |  |  | Number of obs | | = 3046 |
| Part 1: logit |  |  |  |  |  |  |
|  |  |  |  | Number of obs | | = 3046 |
|  |  |  |  | LR chi2 (48) | | = 1191.09 |
|  |  |  |  | Prob > chi2 | | = 0.0000 |
| Log likelihood | = - 1514.3327 |  |  | Pseudo R2 | | = 0.2823 |
| Part 2: glm |  |  |  |  |  |  |
|  |  |  |  | Number of obs | | = 1570 |
| Deviance | = 3709.902027 |  |  | (1/df) Deviance | | = 2.453639 |
| Pearson | = 9644.730825 |  |  | (1/df) Pearson | | = 6.37879 |
| Variance function | : V(u) = u+(1)u^2 | |  | [Neg. Binomial] | |  |
| Link function | : g(u) = ln(u) |  |  | [Log] |  |  |
|  |  |  |  | AIC |  | = 10.68889 |
| Log likelihood |  | = - 8322.776231 | | BIC |  | = - 7416.65 |
| krill | Coef. | Std. Err. | z | P>\|z\| | [95% Conf. Interval] | |
| logit |  |  |  |  |  |  |
| Year_2005 | 0.1143946 | 3.020843 | 0.04 | 0.970 | -5.806349 | 6.035138 |
| Year_2006 | -1.955304 | 3.083389 | -0.63 | 0.526 | -7.998635 | 4.088027 |
| Year_2007 | -8.257875 | 2.866711 | -2.88 | 0.004 | -13.87652 | -2.639225 |
| Year_2008 | -1.870227 | 2.809175 | -0.67 | 0.506 | -7.376108 | 3.635654 |
| Year_2009 | -588.6691 | 102.7765 | -5.73 | 0.000 | -790.1074 | -387.2308 |
| Year_2010 | 3.718564 | 2.634657 | 1.41 | 0.158 | -1.445269 | 8.882396 |
| Year_2011 | -5.966074 | 3.508704 | -1.70 | 0.089 | -12.84301 | 0.91086 |
| Year_2012 | -1.231821 | 3.152489 | -0.39 | 0.696 | -7.410587 | 4.946945 |
| Year_2013 | 18.6145 | 4.615784 | 4.03 | 0.000 | 9.567733 | 27.66127 |
| Month | -12.46733 | 1.604743 | -7.77 | 0.000 | -15.61257 | -9.322089 |
| Month ^2^ | 0.8160945 | 0.1006698 | 8.11 | 0.000 | 0.6187852 | 1.013404 |
| Cell Count (effort) | 0.0064774 | 0.0042849 | 1.51 | 0.131 | -0.0019208 | 0.0148756 |
| SST | 0.4061227 | 0.1890188 | 2.15 | 0.032 | 0.0356528 | 0.7765927 |
| Year_2005 * SST | -0.0901305 | 0.2562024 | -0.35 | 0.725 | -0.592278 | 0.4120171 |
| Year_2006 * SST | 0.2032496 | 0.2260808 | 0.90 | 0.369 | -0.2398606 | 0.6463598 |
| Year_2007 * SST | 0.6290403 | 0.2416775 | 2.60 | 0.009 | 0.1553612 | 1.102719 |
| Year_2008 * SST | -0.4041657 | 0.2445991 | -1.65 | 0.098 | -0.8835711 | 0.0752397 |
| Year_2009 * SST | -0.4544494 | 0.2818051 | -1.61 | 0.107 | -1.006777 | 0.0978784 |
| Year_2010 * SST | -0.2380364 | 0.2279087 | -1.04 | 0.296 | -0.6847293 | 0.2086564 |
| Year_2011 * SST | -0.2139872 | 0.2904621 | -0.74 | 0.461 | -0.7832825 | 0.355308 |
| Year_2012 * SST | -0.5271283 | 0.265563 | -1.98 | 0.047 | -1.047622 | -0.0066344 |
| Year_2013 * SST | -1.138984 | 0.3882185 | -2.93 | 0.003 | -1.899878 | -0.3780897 |
| SSS | 0.9324666 | 0.1846916 | 5.05 | 0.000 | 0.5704777 | 1.294455 |
| SSF | 0.4126723 | 0.0567222 | 7.28 | 0.000 | 0.3014988 | 0.5238458 |
| SSF ^2^ | -0.0137846 | 0.0020581 | -6.70 | 0.000 | -0.0178183 | -0.0097509 |
| Dist. Mainland | 0.0180662 | 0.005243 | 3.45 | 0.001 | 0.0077901 | 0.0283422 |
| Dist. Cordell Bank | 0.0151307 | 0.0025187 | 6.01 | 0.000 | 0.0101942 | 0.0200672 |
| SOI | -1.202816 | 0.1853088 | -6.49 | 0.000 | -1.566014 | -0.8396174 |
| PDO (1-mo lag) | -2.732139 | 0.6102178 | -4.48 | 0.000 | -3.928144 | -1.536134 |
| UI (3-mo lag) | -0.0272949 | 0.0140847 | -1.94 | 0.053 | -0.0549004 | 0.0003105 |
| UI (3-mo lag) ^2^ | 0.0001831 | 0.0000474 | 3.86 | 0.000 | 0.0000902 | 0.0002761 |
| Year_2005 * UI | 0.2453822 | 0.0391279 | 6.27 | 0.000 | 0.1686929 | 0.3220714 |
| Year_2006 * UI | 0.0369741 | 0.059096 | 0.63 | 0.532 | -0.078852 | 0.1528002 |
| Year_2007 * UI | 0.0778272 | 0.0214962 | 3.62 | 0.000 | 0.0356955 | 0.1199589 |
| Year_2008 * UI | 0.0477596 | 0.0156435 | 3.05 | 0.002 | 0.0170989 | 0.0784204 |
| Year_2009 * UI | 9.167357 | 1.571593 | 5.83 | 0.000 | 6.087092 | 12.24762 |
| Year_2010 * UI | 0.0683891 | 0.0153982 | 4.44 | 0.000 | 0.0382093 | 0.098569 |
| Year_2011 * UI | 0.1893751 | 0.023399 | 8.09 | 0.000 | 0.143514 | 0.2352362 |
| Year_2012 * UI | 0.0793541 | 0.0219728 | 3.61 | 0.000 | 0.0362882 | 0.12242 |
| Year_2013 * UI | -0.0581366 | 0.0116132 | -5.01 | 0.000 | -0.080898 | -0.0353752 |
| Year_2005 * UI ^2^ | -0.0013659 | 0.0002324 | -5.88 | 0.000 | -0.0018215 | -0.0009103 |
| Year_2006 * UI ^2^ | -0.0003447 | 0.0002415 | -1.43 | 0.153 | -0.000818 | 0.0001285 |
| Year_2007 * UI ^2^ | -0.0003642 | 0.0000817 | -4.46 | 0.000 | -0.0005244 | -0.0002041 |
| Year_2008 * UI ^2^ | -0.0001736 | 0.0000603 | -2.88 | 0.004 | -0.0002917 | -0.0000554 |
| Year_2009 * UI ^2^ | -0.0340495 | 0.0058318 | -5.84 | 0.000 | -0.0454795 | -0.0226195 |
| Year_2010 * UI ^2^ | -0.0003232 | 0.0000548 | -5.90 | 0.000 | -0.0004305 | -0.0002158 |
| Year_2011 * UI ^2^ | -0.0008764 | 0.0001072 | -8.18 | 0.000 | -0.0010865 | -0.0006663 |
| Year_2012 * UI ^2^ | -0.0003387 | 0.0000752 | -4.51 | 0.000 | -0.000486 | -0.0001914 |
| Year_2013 * UI ^2^ |  |  |  |  |  |  |
| _ Cons | 7.428646 | 8.477054 | 0.88 | 0.381 | -9.186075 | 24.04337 |
| glm |  |  |  |  |  |  |
| Year_2005 | -26.72147 | 9.579211 | -2.79 | 0.005 | -45.49638 | -7.946565 |
| Year_2006 | 7.364914 | 11.69924 | 0.63 | 0.529 | -15.56518 | 30.29501 |
| Year_2007 | -22.46419 | 9.081043 | -2.47 | 0.013 | -40.2627 | -4.66567 |
| Year_2008 | -10.27886 | 9.224513 | -1.11 | 0.265 | -28.35857 | 7.800853 |
| Year_2009 | -17.00503 | 19.11795 | -0.89 | 0.374 | -54.47552 | 20.46546 |
| Year_2010 | -6.042658 | 9.503376 | -0.64 | 0.525 | -24.66893 | 12.58362 |
| Year_2011 | -90.18039 | 20.85657 | -4.32 | 0.000 | -131.0585 | -49.30226 |
| Year_2012 | -24.33919 | 11.85834 | -2.05 | 0.04 | -47.58111 | -1.097266 |
| Year_2013 | -21.32812 | 26.22322 | -0.81 | 0.416 | -72.7247 | 30.06845 |
| Month | -3.710547 | 0.4099678 | -9.05 | 0.000 | -4.514069 | -2.907025 |
| Month ^2^ | 0.2100973 | 0.0250899 | 8.37 | 0.000 | 0.1609219 | 0.2592727 |
| Cell Count (effort) | 0.0101186 | 0.0033793 | 2.99 | 0.003 | 0.0034953 | 0.0167419 |
| SST | 0.5058606 | 1.210847 | 0.42 | 0.676 | -1.867356 | 2.879077 |
| SST ^2^ | 0.0008095 | 0.0486318 | 0.02 | 0.987 | -0.094507 | 0.096126 |
| Year_2005 * SST | 3.49302 | 1.514798 | 2.31 | 0.021 | 0.5240699 | 6.461971 |
| Year_2006 * SST | -1.187477 | 1.824264 | -0.65 | 0.515 | -4.762969 | 2.388014 |
| Year_2007 * SST | 3.103022 | 1.481574 | 2.09 | 0.036 | 0.1991906 | 6.006854 |
| Year_2008 * SST | 1.630832 | 1.528832 | 1.07 | 0.286 | -1.365623 | 4.627287 |
| Year_2009 * SST | 1.497136 | 2.757549 | 0.54 | 0.587 | -3.907561 | 6.901832 |
| Year_2010 * SST | 1.355079 | 1.531435 | 0.88 | 0.376 | -1.646479 | 4.356636 |
| Year_2011 * SST | 15.21102 | 3.26054 | 4.67 | 0.000 | 8.820482 | 21.60156 |
| Year_2012 * SST | 4.453449 | 2.008416 | 2.22 | 0.027 | 0.5170256 | 8.389873 |
| Year_2013 * SST | 3.952317 | 5.068073 | 0.78 | 0.435 | -5.980923 | 13.88556 |
| Year_2005 * SST ^2^ | -0.1441075 | 0.059961 | -2.40 | 0.016 | -0.2616289 | -0.026586 |
| Year_2006 * SST ^2^ | 0.0294426 | 0.0706332 | 0.42 | 0.677 | -0.108996 | 0.1678812 |
| Year_2007 * SST ^2^ | -0.1262331 | 0.0600148 | -2.10 | 0.035 | -0.2438599 | -0.0086063 |
| Year_2008 * SST ^2^ | -0.0639681 | 0.0623908 | -1.03 | 0.305 | -0.1862518 | 0.0583157 |
| Year_2009 * SST ^2^ | -0.0660177 | 0.1032285 | -0.64 | 0.522 | -0.268342 | 0.1363065 |
| Year_2010 * SST ^2^ | -0.0743298 | 0.0612484 | -1.21 | 0.225 | -0.1943745 | 0.0457149 |
| Year_2011 * SST ^2^ | -0.6401423 | 0.1275214 | -5.02 | 0.000 | -0.8900796 | -0.3902051 |
| Year_2012 * SST ^2^ | -0.1915625 | 0.0849054 | -2.26 | 0.024 | -0.357974 | -0.0251509 |
| Year_2013 * SST ^2^ | -0.2054675 | 0.2434196 | -0.84 | 0.399 | -0.6825611 | 0.2716261 |
| SSS | 13.79236 | 5.568633 | 2.48 | 0.013 | 2.878041 | 24.70668 |
| SSS ^2^ | -0.2006893 | 0.0853846 | -2.35 | 0.019 | -0.36804 | -0.0333386 |
| SSF | 0.1257086 | 0.0136089 | 9.24 | 0.000 | 0.0990356 | 0.1523816 |
| Dist. Mainland | 0.0149559 | 0.0069846 | 2.14 | 0.032 | 0.0012664 | 0.0286455 |
| Dist. 200m Iso | 0.105346 | 0.0156295 | 6.74 | 0.000 | 0.0747128 | 0.1359793 |
| Dist. 200m Iso ^2^ | -0.0033288 | 0.0004443 | -7.49 | 0.000 | -0.0041996 | -0.0024579 |
| Dist. Cordell Bank | -0.0303305 | 0.0087988 | -3.45 | 0.001 | -0.0475758 | -0.0130852 |
| Dist. Cordell Bank ^2^ | 0.0003298 | 0.0000832 | 3.96 | 0.000 | 0.0001667 | 0.0004929 |
| Dist. SEFI | -0.0103087 | 0.004776 | -2.16 | 0.031 | -0.0196694 | -0.0009479 |
| Average Depth | -0.0016252 | 0.0011141 | -1.46 | 0.145 | -0.0038087 | 0.0005583 |
| Average Depth ^2^ | -1.38E-06 | 1.72E-06 | -0.80 | 0.423 | -4.76E-06 | 2.00E-06 |
| Average Depth ^3^ | 1.99E-10 | 6.94E-10 | 0.29 | 0.774 | -1.16E-09 | 1.56E-09 |
| Contour Index | 1.626499 | 0.6320889 | 2.57 | 0.010 | 0.3876273 | 2.86537 |
| Contour Index ^2^ | -1.59695 | 0.7670236 | -2.08 | 0.037 | -3.100289 | -0.0936113 |
| NPGO (3-mo lag) | -1.690843 | 0.2054305 | -8.23 | 0.000 | -2.09348 | -1.288207 |
| NPGO (3-mo lag) ^2^ | 0.5536906 | 0.1426907 | 3.88 | 0.000 | 0.274022 | 0.8333592 |
| UI (3-mo lag) | 0.0057209 | 0.0015744 | 3.63 | 0.000 | 0.0026351 | 0.0088068 |
| Year_2005 * UI | 0.0051671 | 0.0025578 | 2.02 | 0.043 | 0.0001538 | 0.0101803 |
| Year_2006 * UI | 0.0087971 | 0.0019937 | 4.41 | 0.000 | 0.0048895 | 0.0127046 |
| Year_2007 * UI | 0.0088964 | 0.0023106 | 3.85 | 0.000 | 0.0043677 | 0.0134252 |
| Year_2008 * UI | -0.0054839 | 0.002208 | -2.48 | 0.013 | -0.0098115 | -0.0011563 |
| Year_2009 * UI | 0.0676228 | 0.0125451 | 5.39 | 0.000 | 0.0430348 | 0.0922107 |
| Year_2010 * UI | 0.0059767 | 0.0017393 | 3.44 | 0.001 | 0.0025676 | 0.0093857 |
| Year_2011 * UI | 0.0161231 | 0.0033039 | 4.88 | 0.000 | 0.0096476 | 0.0225985 |
| Year_2012 * UI | -0.0048529 | 0.0016558 | -2.93 | 0.003 | -0.0080981 | -0.0016076 |
| Year_2013 * UI | 0.0175237 | 0.0058731 | 2.98 | 0.003 | 0.0060126 | 0.0290348 |
| _ Cons | -225.0169 | 90.6551 | -2.48 | 0.013 | -402.6976 | -47.33613 |
