# Supplementary material for: Spatial Distribution and Temporal Patterns of Cassin’s Auklet Foraging and Their Euphausiid Prey in a Variable Ocean Environment
Source: PLoS One. 2015 Dec 2;10(12):e0144232. doi: 10.1371/journal.pone.0144232 (PMC4668006; doi:10.1371/journal.pone.0144232)
Supplement: S2 Table — Model output assumes base year is 2004. (DOCX) [file pone.0144232.s002.docx]

S2 Table. Coefficients, standard errors, z values, p values, and associated 95% confidence intervals for all quantitative variables (including years and interactions with years) for the zero-inflated negative binomial regression model for Cassin’s auklets. Model output assumes base year is 2004.

| Zero-inflated negative binomial regression | | |  | Number of obs | | = 3879 |
| --- | --- | --- | --- | --- | --- | --- |
|  |  |  |  | Nonzero obs | | = 832 |
|  |  |  |  | Zero obs | | = 3047 |
| Inflation model |  | = logit |  | LR chi2 (35) | | = 818.39 |
| Log likelihood |  | = -4420.639 |  | Prob > chi2 | | = 0.0000 |
| Cassin’s auklet | Coef. | Std. Err. | z | P>\|z\| | [95% Conf. Interval] | |
| Cassin’s auklet |  |  |  |  |  |  |
| Year_2005 | 155.5994 | 41.17989 | 3.78 | 0.000 | 74.8883 | 236.3105 |
| Year_2006 | 253.2461 | 35.47579 | 7.14 | 0.000 | 183.7148 | 322.7774 |
| Year_2007 | 199.7966 | 41.29645 | 4.84 | 0.000 | 118.8571 | 280.7362 |
| Year_2008 | 96.98992 | 33.10826 | 2.93 | 0.003 | 32.09893 | 161.8809 |
| Year_2009 | -81.55508 | 49.63897 | -1.64 | 0.100 | -178.8457 | 15.73552 |
| Year_2010 | -15.84714 | 36.67799 | -0.43 | 0.666 | -87.73467 | 56.04039 |
| Year_2011 | 160.2504 | 38.1152 | 4.2 | 0.000 | 85.54601 | 234.9549 |
| Year_2012 | 67.55212 | 39.54002 | 1.71 | 0.088 | -9.944891 | 145.0491 |
| Year_2013 | 140.7279 | 31.19172 | 4.51 | 0.000 | 79.59323 | 201.8625 |
| Month | 0.4213482 | 0.5126992 | 0.82 | 0.411 | -0.5835238 | 1.42622 |
| Month ^2^ | -0.0348403 | 0.0348667 | -1.00 | 0.318 | -0.1031778 | 0.0334971 |
| SSS | 127.2298 | 35.5615 | 3.58 | 0.000 | 57.5305 | 196.929 |
| SSS ^2^ | -1.856875 | 0.5337374 | -3.48 | 0.001 | -2.902981 | -0.8107687 |
| Year_2005 * SSS | -4.647781 | 1.238245 | -3.75 | 0.000 | -7.074696 | -2.220866 |
| Year_2006 * SSS | -7.642804 | 1.063189 | -7.19 | 0.000 | -9.726617 | -5.558992 |
| Year_2007 * SSS | -5.994323 | 1.23943 | -4.84 | 0.000 | -8.423562 | -3.565084 |
| Year_2008 * SSS | -2.839541 | 0.9910867 | -2.87 | 0.004 | -4.782035 | -0.8970465 |
| Year_2009 * SSS | 2.547385 | 1.494063 | 1.71 | 0.088 | -0.3809244 | 5.475695 |
| Year_2010 * SSS | 0.5335533 | 1.097513 | 0.49 | 0.627 | -1.617534 | 2.68464 |
| Year_2011 * SSS | -4.732991 | 1.143389 | -4.14 | 0.000 | -6.973992 | -2.49199 |
| Year_2012 * SSS | -1.980323 | 1.183486 | -1.67 | 0.094 | -4.299912 | 0.3392667 |
| Year_2013 * SSS | -4.139342 | 0.9356833 | -4.42 | 0.000 | -5.973247 | -2.305436 |
| Dist. SEFI | 0.0296437 | 0.0041217 | 7.19 | 0.000 | 0.0215653 | 0.0377222 |
| Dist. 200m Iso | 0.0794521 | 0.0425744 | 1.87 | 0.062 | -0.0039923 | 0.1628965 |
| Dist. 200m Iso ^2^ | -0.0088576 | 0.0014267 | -6.21 | 0.000 | -0.0116539 | -0.0060612 |
| Average Depth | 0.0122394 | 0.0024687 | 4.96 | 0.000 | 0.007401 | 0.0170779 |
| Average Depth ^2^ | 0.0000146 | 4.16E-06 | 3.51 | 0.000 | 6.44E-06 | 0.0000227 |
| Average Depth ^3^ | 5.00E-09 | 1.74E-09 | 2.88 | 0.004 | 1.60E-09 | 8.40E-09 |
| Contour Index | 7.152712 | 1.279647 | 5.59 | 0.000 | 4.64465 | 9.660774 |
| Contour Index ^2^ | -5.062889 | 1.475388 | -3.43 | 0.001 | -7.954598 | -2.171181 |
| SOI (1-mo lag) | -0.9207524 | 0.1177089 | -7.82 | 0.000 | -1.151458 | -0.6900472 |
| SOI (1-mo lag) ^2^ | 0.3060688 | 0.0419646 | 7.29 | 0.000 | 0.2238197 | 0.3883179 |
| PDO (3-mo lag) | 0.4923281 | 0.2242361 | 2.20 | 0.028 | 0.0528334 | 0.9318229 |
| PDO (3-mo lag) ^2^ | -1.338759 | 0.1582932 | -8.46 | 0.000 | -1.649008 | -1.02851 |
| UI (2-mo lag) | -0.0037098 | 0.0015688 | -2.36 | 0.018 | -0.0067846 | -0.000635 |
| _ Cons | -2178.475 | 592.5751 | -3.68 | 0.000 | -3339.901 | -1017.049 |
| Log (Area) | 1 | (offset) |  |  |  |  |
| Inflate |  |  |  |  |  |  |
| Seastate | 0.4259129 | 0.1412156 | 3.02 | 0.003 | 0.1491354 | 0.7026905 |
| Visibility | 0.6216373 | 0.191325 | 3.25 | 0.001 | 0.2466472 | 0.9966274 |
| _ Cons | -7.100618 | 1.763031 | -4.03 | 0.000 | -10.5561 | -3.645141 |
| /lnalpha | 2.130516 | 0.065361 | 32.60 | 0.000 | 2.002411 | 2.258621 |
| alpha | 8.419211 | 0.550288 |  |  | 7.406892 | 9.569887 |
| Likelihood-ratio test of alpha=0: | | chibar2(01) = 2.2e+04 | |  | Pr>chibar2 | = 0.0000 |
| Vuong test of zinb vs. standard negative binomial: | | | z = 2.63 |  | Pr>z | = 0.0043 |
